# Genotype–phenotype correlations for *COL4A3*–*COL4A5* variants resulting in Gly substitutions in Alport syndrome

**DOI:** 10.1038/s41598-022-06525-9

**Published:** 2022-02-17

**Authors:** Joel T. Gibson, Mary Huang, Marina Shenelli Croos Dabrera, Krushnam Shukla, Hansjörg Rothe, Pascale Hilbert, Constantinos Deltas, Helen Storey, Beata S. Lipska-Ziętkiewicz, Melanie M. Y. Chan, Omid Sadeghi-Alavijeh, Daniel P. Gale, Genomics England Research Consortium, J. C. Ambrose, P. Arumugam, E. L. Baple, M. Bleda, F. Boardman-Pretty, J. M. Boissiere, C. R. Boustred, H. Brittain, M. J. Caulfield, G. C. Chan, C. E. H. Craig, L. C. Daugherty, A. de Burca, A. Devereau, G. Elgar, R. E. Foulger, T. Fowler, P. Furió-Tarí, A. Giess, J. M. Hackett, D. Halai, A. Hamblin, S. Henderson, J. E. Holman, T. J. P. Hubbard, K. Ibáñez, R. Jackson, L. J. Jones, D. Kasperaviciute, M. Kayikci, A. Kousathanas, L. Lahnstein, K. Lawson, S. E. A. Leigh, I. U. S. Leong, F. J. Lopez, F. Maleady-Crowe, J. Mason, E. M. McDonagh, L. Moutsianas, M. Mueller, N. Murugaesu, A. C. Need, C. A. Odhams, A. Orioli, C. Patch, D. Perez-Gil, M. B. Pereira, D. Polychronopoulos, J. Pullinger, T. Rahim, A. Rendon, P. Riesgo-Ferreiro, T. Rogers, M. Ryten, K. Savage, K. Sawant, R. H. Scott, A. Siddiq, A. Sieghart, D. Smedley, K. R. Smith, S. C. Smith, A. Sosinsky, W. Spooner, H. E. Stevens, A. Stuckey, R. Sultana, M. Tanguy, E. R. A. Thomas, S. R. Thompson, C. Tregidgo, A. Tucci, E. Walsh, S. A. Watters, M. J. Welland, E. Williams, K. Witkowska, S. M. Wood, M. Zarowiecki, Agne Cerkauskaite, Judy Savige

**Affiliations:** 1 Department of Medicine (Melbourne Health and Northern Health), Royal Melbourne Hospital, The University of Melbourne, Parkville, VIC 3050 Australia; 2 Centre for Nephrology and Metabolic Disorders, 02943 Weisswasser, Germany; 3 Departement de Biologie Moleculaire, Institute de Pathologie et de Genetique ASBL, Gosselies, Belgium; 4 Center of Excellence in Biobanking and Biomedical Research, University of Cyprus Medical School, Nicosia, Cyprus; 5 Molecular Genetics, Viapath Laboratories, 5th Floor Tower Wing, Guy’s Hospital, London, SE1 9RT UK; 6 Centre for Rare Diseases, and Clinical Genetics Unit, Medical University of Gdańsk, Gdańsk, Poland; 7 Department of Renal Medicine, University College London, London, UK; 8 Institute of Biomedical Sciences, Faculty of Medicine, Vilnius University, Vilnius, Lithuania; 9 Genomics England, London, UK; 10 William Harvey Research Institute, Queen Mary University of London, London, EC9M 6BQ UK

**Keywords:** Genetics, Diseases, Nephrology

## Abstract

Alport syndrome is the commonest inherited kidney disease and nearly half the pathogenic variants in the *COL4A3–COL4A5* genes that cause Alport syndrome result in Gly substitutions. This study examined the molecular characteristics of Gly substitutions that determine the severity of clinical features. Pathogenic *COL4A5* variants affecting Gly in the Leiden Open Variation Database in males with X-linked Alport syndrome were correlated with age at kidney failure (n = 157) and hearing loss diagnosis (n = 80). Heterozygous pathogenic *COL4A3* and *COL4A4* variants affecting Gly (n = 304) in autosomal dominant Alport syndrome were correlated with the risk of haematuria in the UK 100,000 Genomes Project. Gly substitutions were stratified by exon location (1 to 20 or 21 to carboxyl terminus), being adjacent to a non-collagenous region (interruption or terminus), and the degree of instability caused by the replacement residue. Pathogenic *COL4A5* variants that resulted in a Gly substitution with a highly destabilising residue reduced the median age at kidney failure by 7 years (p = 0.002), and age at hearing loss diagnosis by 21 years (p = 0.004). Substitutions adjacent to a non-collagenous region delayed kidney failure by 19 years (p = 0.014). Heterozygous pathogenic *COL4A3* and *COL4A4* variants that resulted in a Gly substitution with a highly destabilising residue (Arg, Val, Glu, Asp, Trp) were associated with an increased risk of haematuria (p = 0.018), and those adjacent to a non-collagenous region were associated with a reduced risk (p = 0.046). Exon location had no effect. In addition, *COL4A5* variants adjacent to non-collagenous regions were over-represented in the normal population in gnomAD (p < 0.001). The nature of the substitution and of nearby residues determine the risk of haematuria, early onset kidney failure and hearing loss for Gly substitutions in X-linked and autosomal dominant Alport syndrome.

## Introduction

Alport syndrome (AS) is an inherited basement membrane disease characterised by progressive kidney failure, sensorineural hearing loss and ocular abnormalities^1^. Estimates of its disease frequency range from a prevalence of one in 5000 people in Utah^2^ to one in 53,000 live births in Finland^3^, but the number of people with a predicted genetic risk of disease is even higher^4^.

AS results from pathogenic variants in *COL4A5*^5^, *COL4A3* or *COL4A4*^6^. These genes encode the collagen IV α5, α3 and α4 chains respectively, that trimerise to form a triple helical structure and chickenwire network typical of basement membranes^7,8^. X-Linked AS is the commonest form that causes kidney failure^9^. Males are more severely affected than females and 70% have kidney failure by the age of 30^10^. Females are affected twice as often as males but normally have a milder and more variable phenotype^11^ due in part to non-random X- chromosome inactivation^12^.

Autosomal recessive AS is less common, and results from two pathogenic variants affecting the *COL4A3* or *COL4A4* genes^6^. These may be homozygous^6^, or compound heterozygous variants *in trans*^13^*.* Digenic variants result most often from one pathogenic variant in *COL4A3* and one in *COL4A4*^14^. Pathogenic heterozygous variants in *COL4A3* or *COL4A4* result in autosomal dominant AS (also known as ‘thin basement membrane nephropathy’), with haematuria^15^, and late-onset kidney failure in up to 25% of affected individuals in hospital-based series^16–18^.

Each collagen IV α chain comprises an intermediate collagenous domain of Gly Xaa Yaa triplet repeats^19–21^. However, the collagen IV α chains differ from most other collagen types in that the collagenous domain includes 21–26 short non-collagenous interruptions^21^. These provide flexibility to an otherwise rigid molecule, and may include important ligand binding sites^22^. Collagen IV chains also differ in that the mature chains retain their non-collagenous amino and carboxyl termini, which interact with the termini of neighbouring trimers to create the network^8^. The carboxyl terminus is also the site of chain recognition where trimerisation begins, proceeding in a zipper-like manner towards the amino terminus^23^.

Previous genotype–phenotype correlations in males with pathogenic *COL4A5* variants have demonstrated that truncating variants and large deletions lead to the most severe phenotype, with the youngest age at kidney failure^10,24^. The corresponding mRNA is degraded by the podocytes, the and there is no collagen IV α5 chain incorporated into the trimer or staining in a kidney biopsy^25^. Missense variants usually result in milder disease^10,24^ and the abnormal chain may be incorporated into mature trimers that are secreted into the basement membrane^25^ in reduced amounts^26^. The abnormal chains are also often retained within the podocytes, activating the unfolded protein response and increasing endoplasmic reticulum stress^27,28^. Similar studies in females with heterozygous *COL4A5* variants have not found such a clear genotype–phenotype correlation with age at kidney failure^11,12^, but a recent study suggested that females with missense variants were less likely to develop proteinuria and had better kidney function than those with other variant types^29^.

In autosomal recessive AS, individuals with at least one truncating variant in *COL4A3* or *COL4A4* are more likely to progress to kidney failure before the age of 30 years than those with non-truncating variants^30^. Disease progression also correlates with the number of missense variants, where individuals with at least one missense variant have a delayed onset of kidney failure and hearing loss compared with those with none^31^. In individuals with autosomal dominant AS, heterozygous truncating variants in *COL4A3* or *COL4A4* are associated with an earlier age at kidney failure than those with missense variants^16^.

Missense variants affecting Gly residues in the collagenous Gly Xaa Yaa repeats are the commonest pathogenic type^32^. These residues are critical to the structure since Gly is the only amino acid small enough to fit within the core of triple helix and allow close packing of the chains^32,33^. Substitution with any other amino acid may destabilise the trimer, interfere with triple helix propagation, and cause disease^34^.

The clinical phenotype associated with pathogenic Gly missense variants is highly variable. Gly substitution with a bulky or charged amino acid usually leads to more severe clinical features^35^, but contrary examples also exist^36^. In other collagen types, Gly substitutions in the amino exons result in a milder phenotype^37^, but evidence for this in collagen IV is conflicting^24,38^. Location adjacent to a non-collagenous interruption has also been associated with a milder phenotype^39^, but again this is variable^35^.

The aim of this study was to better understand the molecular features of Gly substitutions in the *COL4A3–COL4A5* genes that affect disease severity.

## Methods

### Variant databases

Three variant databases were examined. The Leiden Open Variation Database (LOVD) is an open source database of genomic variants with associated phenotypes (https://www.lovd.nl)^40^. It includes variants published in the literature in addition to those submitted directly by laboratories, and has recently been updated to include a total of 3869 (including 2988 pathogenic) *COL4A5* variants. Pathogenicity was assessed by the submitting laboratory, or where none was provided, using the VarSome scores (https://varsome.com) based on the American College of Medical Genetics and Genomics and Association for Molecular Pathology (ACMG/AMP) criteria. Varsome scores automatically include previous ClinVar or other published assessments (PP5). Many variants also included clinical data such as gender, age at kidney failure, hearing loss and ocular abnormalities. This database was used to determine whether molecular features of pathogenic *COL4A5* variants that resulted in Gly substitutions affected age at kidney failure or hearing loss diagnosis using survival analysis.

The Genomics England 100,000 Genomes Project (100kGP) is a database comprising genomic and clinical data from individuals and families with various diseases, including familial haematuria and other inherited kidney disease (https://www.genomicsengland.co.uk; version 10 data release)^41^. This was used to determine whether molecular features of heterozygous pathogenic *COL4A3* and *COL4A4* variants that resulted in Gly substitutions were associated with haematuria.

The Genome Aggregation Database (gnomAD) comprises exomes and genomes from individuals recruited as part of various disease-specific and population genetic studies (gnomAD version 2.1.1; https://gnomad.broadinstitute.org; accessed 11 September 2021)^42^. These primarily include participants and controls from studies of cardiovascular disease, diabetes or psychiatric disorders, who have not been selected for kidney disease but rather to represent a cross-section of the population. This database was used to determine whether milder molecular characteristics of *COL4A5* Gly substitutions were increased the general population.

The individuals whose variants and other deidentified information were included in these databases had provided informed consent at the time of recruitment under the supervision of the corresponding institutional review boards.

### Reference sequences

*COL4A5* variants were described using the collagen IV α5 chain isoform 2 reference sequence comprising 53 exons (NM_033380.3). *COL4A3* and *COL4A4* variants were described using the reference sequences for the collagen IV α3 (NM_000091.5) and α4 (NM_000092.5) chains respectively.

### Predicted splicing changes

Previous studies have demonstrated that exonic nucleotide substitutions affecting Gly codons in *COL4A5* sometimes result in abnormal splicing^43,44^, and recent evidence suggests that this is common when the affected base is the final nucleotide of an exon^45^. To ensure that unknown splicing variants were not unintentionally included in this study, all variants occurring within 3 bases of a splice site were analysed using MaxEntScan to determine whether they were likely to affect normal splicing^46^. MaxEntScan was chosen since it is freely available and was able to correctly identify 6 known exonic splice changes previously reported for *COL4A5* (data not shown). Both mutant and wild type sequences were scored using the maximum entropy model. Variants were considered to affect splicing where the mutant score was more than 15% lower than the wild type score^47^. All variants predicted to affect normal splicing were excluded to ensure that any phenotypic effect attributed to a variant was due solely to a missense change, rather than an unreported splicing change (Supplemental Table 1).

### Molecular characteristics

Three molecular features were examined for each Gly substitution. These were the molecular location of the variant (exons 1 to 20, or exons 21 to carboxyl terminus), whether the variant was adjacent to a non-collagenous interruption or terminus (also called ‘non-collagenous boundary’ variants) (Supplemental Table 2), and the degree of instability caused by the residue replacing Gly (Ala, Ser, Cys were considered mildly destabilising; Arg, Val, Glu, Asp, Trp were considered highly destabilising)^34^.

**Figure 1:**
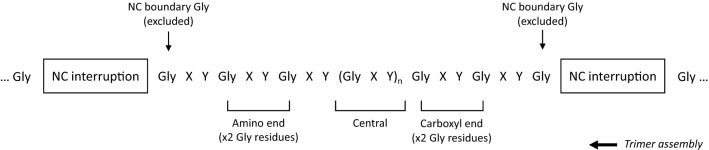
Terminology used in this study to describe the position of Gly residues with respect to their local collagenous region. One single ‘local’ collagenous region is shown, bounded by non-collagenous interruptions at either end. The boxes could also represent either of the two non-collagenous termini. The non-collagenous boundary Gly residues are a special case, so were not included in this subgroup. The larger arrow indicates the direction of trimerisation, which is initiated at the carboxyl terminus and then proceeds in the amino direction. NC, non-collagenous.

In addition a subgroup of variants was examined separately to determine the effect of a variant’s relative location with respect to its local collagenous region (a single uninterrupted stretch of Gly XY repeats flanked by two non-collagenous interruptions/termini). This subgroup excluded all non-collagenous boundary variants to ensure that the significantly different phenotypes usually observed for these variants did not obscure any small effect size. The remaining variants were considered in three groups: variants affecting the 2 Gly residues at the amino end of a local collagenous region (not counting the boundary Gly), variants affecting the 2 Gly residues at the carboxyl end of a local collagenous regions, and all other variants falling between these two ends (Fig. 1).

#### *COL4A5*

##### Kidney failure

*COL4A5* variants reported in LOVD were filtered to include those with entries including any of the following keywords: ‘renal’, ‘failure’, ‘ESRD’, ‘ESRF’, ‘ESKD’, ‘ESKF’, ‘transplant’, ‘dialysis’ (Supplemental Fig. 1a). Only variants affecting a Gly residue in a collagenous sequence (GlyXaaYaa) and reported as ‘Pathogenic’ or ‘Likely Pathogenic’ were included. Predicted splicing variants were excluded as described. Each entry was then examined manually to determine the age and kidney failure status of male participants. Age at kidney failure was defined as the age at diagnosis of kidney failure, or where this was not reported, the age at commencement of dialysis, or at first kidney transplant. Unclear or ambiguous entries in LOVD were resolved by referring to the original manuscripts.

Each family was included once only. Where multiple affected males were reported in the same family, the mean age at kidney failure was used (or median age at kidney failure, if this was the only value available). Where only a range of ages for kidney failure was reported, the midpoint of this range was used. Where a male had not yet progressed to kidney failure, the age of the male at the most recent report was used as a censored data point. Families with only affected females, or participants who had multiple variants in the *COL4A3*-*COL4A5* genes were excluded.

##### Hearing loss

*COL4A5* variants reported in LOVD were filtered to include those which had entries with the following keywords: ‘hearing’, ‘hypoacusia’, ‘deaf’, ‘sensorineural’, or ‘audio’ (Supplemental Fig. 1b). Onset of hearing loss is often poorly recognised and probably occurs much earlier than reported, so here the age at diagnosis of hearing loss was used as a measure of hearing loss severity. Ages were extracted as for kidney failure, using similar inclusion and exclusion criteria.

#### *COL4A3*/*COL4A4*

##### Haematuria

Individuals with and without haematuria were identified from the 100kGP database. The haematuria cohort included unrelated individuals with any haematuria-related terms (HP:0000790, hematuria; HP:0002907, microscopic hematuria; HP:0012587, macroscopic hematuria), excluding those with a diagnosis of kidney or bladder cancer, or other less common causes (n = 2221). The ancestry-matched control cohort included all individuals who did not have any documented haematuria in their medical records (n = 37,200). Individuals were then filtered to include only those reported to have a heterozygous Gly missense variant in *COL4A3* or *COL4A4*. Variants near splice sites were assessed for predicted splice changes as described.

All variants included were assessed for the same molecular characteristics as described above, and a logistic regression model used to identify which features were associated with a difference in risk for haematuria. All variants not adjacent to a non-collagenous region were also examined separately to determine any associations with local collagenous location.

### Prevalence of *COL4A5* non-collagenous boundary variants in normals

To determine whether non-collagenous boundary variants were increased in the general population, the collagenous location and prevalence of all Gly substitutions in gnomAD were investigated. Predicted splicing variants were excluded.

The expected proportion of variants affecting non-collagenous boundary Gly variants was calculated using a neighbour-dependent substitution rate model^48^. This model was chosen since it takes into account the higher rates of substitution seen for transitions than transversions, as well as the effect of the neighbouring nucleotides. This model has also been used previously in the context of Gly substitutions in collagen molecules^34^. The expected proportion of variants affecting non-collagenous boundary Gly residues was compared with the proportion observed in gnomAD. The population prevalence of individuals with these variants was then calculated based on the allele frequencies reported in gnomAD, correcting for numbers reported in homozygous females.

### Statistical analysis

All statistical analyses were performed using R (version 3.6.2). Survival analysis was performed using the *survival* package^49,50^. Separate survival curves were produced for each molecular feature using the Kaplan–Meier method, and compared using the log-rank test. The individual contribution of each covariate was then analysed using a Cox proportional hazards model. The overall significance of this model was assessed using the likelihood ratio test.

Logistic regression models were used to examine the associations between the molecular features and haematuria. The proportion of variance in haematuria explained by the model was assessed using McFadden’s pseudo-R^2^ and calculated using the *blorr* package^51^.

Expected and observed proportions of variants in gnomAD were compared with the exact binomial test. For all analyses, a p-value less than 0.05 was considered significant. Figures were produced using the *survminer*^52^ and *forestplot*^53^ packages.

## Results

### *COL4A5*

#### Kidney failure

**Figure 2:**
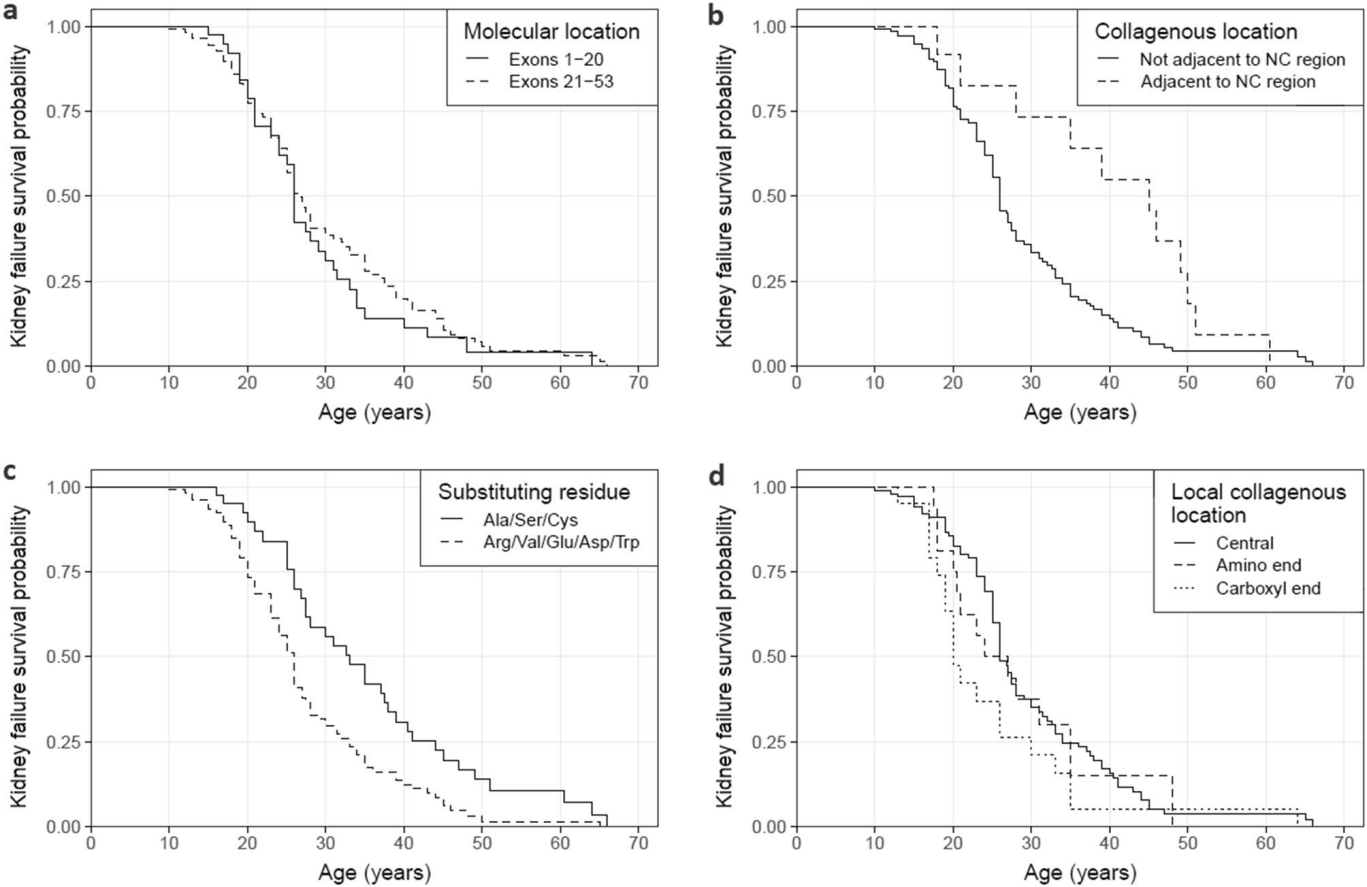
Proportion of cases without kidney failure for *COL4A5* Gly missense variants reported in LOVD. Variants were stratified by (**a**) molecular location (p = 0.41), (**b**) collagenous location (p = 0.014) and (**c**) substituting residue (p = 0.002). (**d**) Excluding variants affecting NC boundary residues, variants were further stratified by relative location within their local collagenous region (p = 0.14). Censored data points are not shown. NC, non-collagenous.

**Figure 3:**
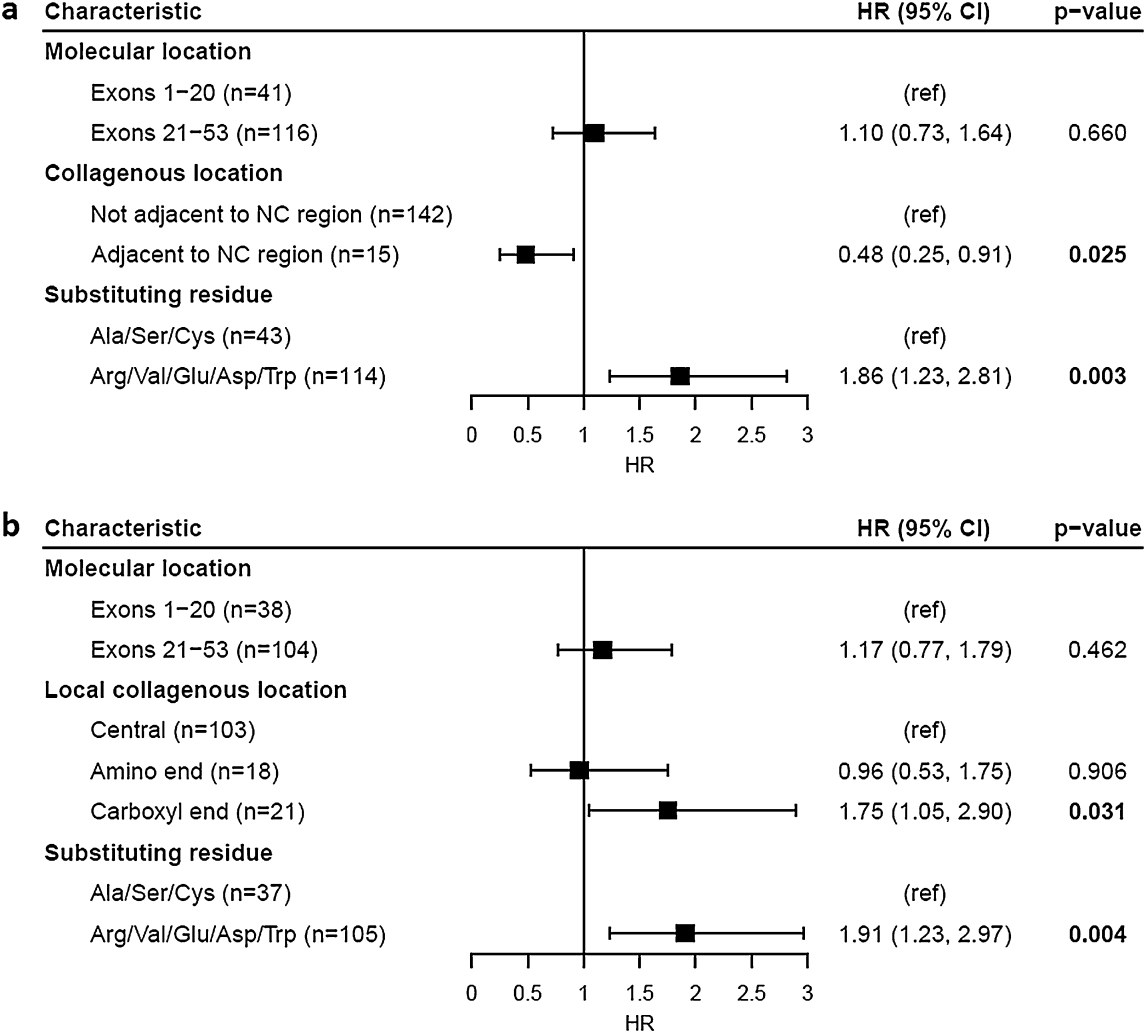
Cox proportional hazards model of kidney failure risk for *COL4A5* Gly missense variants reported in LOVD. Analysis was performed for (**a**) all Gly missense variants (overall significance of model, p < 0.001) and (**b**) excluding NC boundary Gly variants (overall significance of model, p = 0.014). HR (95% CI), Hazard ratio (95% confidence interval); NC, non-collagenous; ref, Reference group.

One hundred and fifty-seven families were studied, including 129 with at least one male with kidney failure (Table 1a, Fig. 2a–c). Overall, the median age at kidney failure was 26 years (95% CI 25–28). Age at kidney failure did not differ for variants located within the first 20 exons compared with those in exons 21 to 53 (p = 0.41). Substitution of a non-collagenous boundary Gly residue delayed median time to kidney failure by 19 years compared with those not adjacent to a non-collagenous region (p = 0.014), while substitution with a highly destabilising residue shortened median time to kidney failure by 7 years compared with mildly destabilising residues (p = 0.002). Substitution of a non-collagenous boundary Gly residue was independently associated with a decreased risk of kidney failure (p = 0.025), while substitution with a highly destabilising residue was independently associated with an increased risk (p = 0.003) (Fig. 3a).

**Table 1:** Median age at kidney failure of *COL4A5* Gly missense variants reported in LOVD for each molecular characteristic.

	N kidney failure	Median age at kidney failure (years) (95% CI)	p-value
**a. All variants (n = 157)**			
Molecular location			
Exons 1–20 (n = 41)	35	26 (24, 31)	0.41
Exons 21–53 (n = 116)	94	26.8 (25, 30)	
Collagenous location			
Not adjacent to NC region (n = 142)	118	26 (25, 27.5)	**0.014**
Adjacent to NC region (n = 15)	11	45 (35, ND)	
Substituting residue			
Ala/Ser/Cys (n = 43)	35	33 (27.5, 40.5)	**0.002**
Arg/Val/Glu/Asp/Trp (n = 114)	94	26 (24, 27)	
**b. Excluding NC boundary variants (n = 142)**			
Local collagenous location			
Central (n = 103)	85	26 (26, 29)	0.14
Amino end (n = 18)	14	25.5 (20.5, ND)	
Carboxyl end (n = 21)	19	20 (19, 33)	

NC, non-collagenous; ND, not done (too few data); 95% CI, 95% confidence interval.

Significant values are in bold.

Considering only the subgroup excluding the non-collagenous boundary variants, location within a local collagenous region did not affect the median time to kidney failure (p = 0.14) (Table 1b, Fig. 2d). However, substitution of a Gly residue at the carboxyl end of a local collagenous region was independently associated with an increased risk of kidney failure compared with substitutions in the central region (p = 0.031) (Fig. 3b). Substitution with a highly destabilising residue remained significantly associated with an increased risk of kidney failure for this subgroup (p = 0.004).

#### Hearing loss

**Figure 4:**
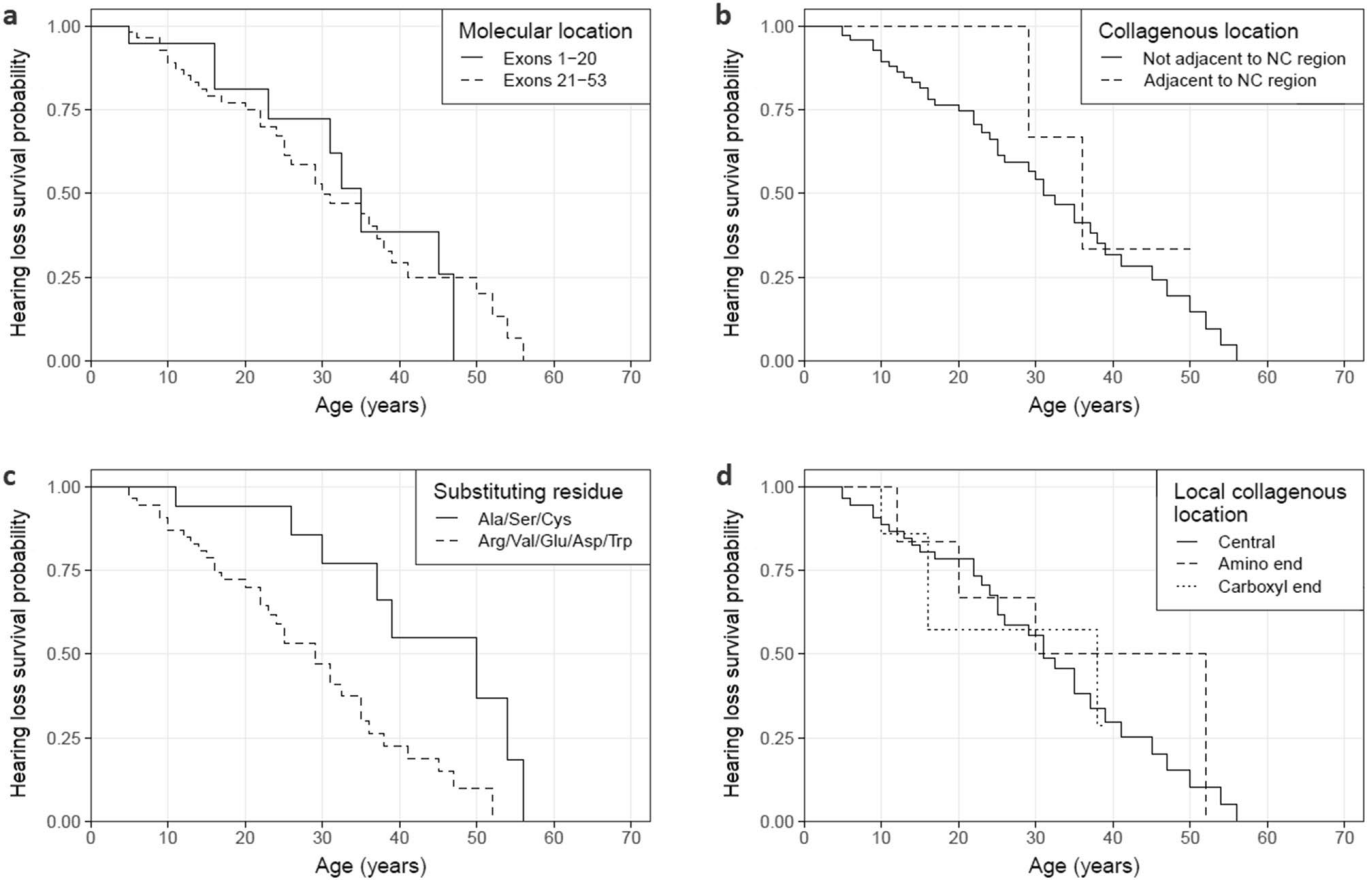
Proportion of cases without a hearing loss diagnosis for *COL4A5* Gly missense variants reported in LOVD. Variants were stratified by (**a**) molecular location (p = 0.85), (**b**) collagenous location (p = 0.38) and (**c**) substituting residue (p = 0.004). (**d**) Excluding variants affecting NC boundary residues, variants were further stratified by relative location within their local collagenous region (p = 0.77). Censored data points are not shown. NC, non-collagenous.

**Figure 5:**
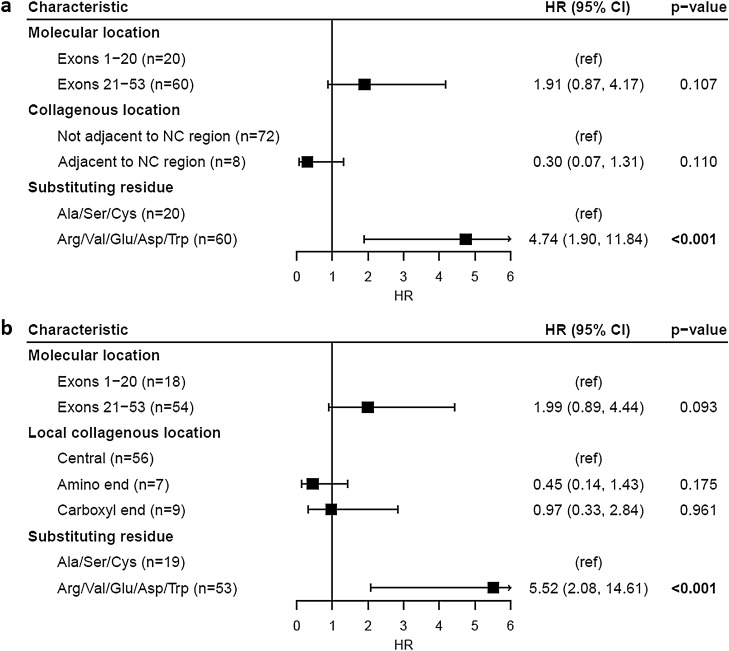
Cox proportional hazards model of hearing loss diagnosis risk for *COL4A5* Gly missense variants reported in LOVD. Analysis was performed for (**a**) all Gly missense variants (overall significance of model, p = 0.002) and (**b**) excluding NC boundary Gly variants (overall significance of model, p = 0.004). HR (95% CI), Hazard ratio (95% confidence interval); NC, non-collagenous; ref, reference group.

Eighty families were studied, including 42 with at least one report of hearing loss in a male (Table 2a, Fig. 4a–c). Median age at hearing loss diagnosis did not differ for variants located within the first 20 exons compared with those located in exons 21 to 53 (p = 0.85). Unlike with kidney failure, median age at hearing loss diagnosis did not differ between variants affecting non-collagenous boundary Gly residues and variants not adjacent to a non-collagenous region (p = 0.38). However, the sample size for the non-collagenous boundary variants was small (n = 8), and only 2 of these eight families had a report of hearing loss at the time of the study. Substitution with a highly destabilising residue shortened median time to diagnosis of hearing loss by 21 years compared with mildly destabilising residues (p = 0.004), and this was the only molecular feature independently associated with an increased risk of hearing loss (p < 0.001) (Fig. 5a).

**Table 2:** Median age at hearing loss diagnosis of *COL4A5* Gly missense variants reported in LOVD for each molecular characteristic.

	N hearing loss	Median age at hearing loss diagnosis (years) (95% CI)	p-value
**a. All variants (n = 80)**			
Molecular location			
Exons 1–20 (n = 20)	9	35 (31, ND)	0.85
Exons 21–53 (n = 60)	33	30 (25, 41)	
Collagenous location			
Not adjacent to NC region (n = 72)	40	31 (25, 41)	0.38
Adjacent to NC region (n = 8)	2	36 (29, ND)	
Substituting residue			
Ala/Ser/Cys (n = 20)	8	50 (37, ND)	**0.004**
Arg/Val/Glu/Asp/Trp (n = 60)	34	29 (23, 36)	
**b. Excluding NC boundary variants (n = 72)**			
Local collagenous location			
Central (n = 56)	32	31 (25, 41)	0.77
Amino end (n = 7)	4	41 (20, ND)	
Carboxyl end (n = 9)	4	38 (16, ND)	

NC, non-collagenous; ND, not done (too few data); 95% CI, 95% confidence interval.

Significant values are in bold.

Excluding all non-collagenous boundary variants, location within a local collagenous region did not affect the median time to hearing loss diagnosis (p = 0.77) (Table 2b, Fig. 4d). Risk of hearing loss also did not differ for substitutions at the amino (p = 0.18) or carboxyl ends (p = 0.96) (Fig. 5b). Substitution with a highly destabilising residue remained significantly associated with an increased risk of hearing loss for this subgroup (p < 0.001).

### *COL4A3*/*COL4A4*

#### Haematuria

This cohort comprised 304 individuals from the 100kGP, including 48 with documented haematuria (Table 3). In total, 153 unique heterozygous *COL4A3* and *COL4A4* Gly missense variants were studied, and most (n = 105) were found once only.

**Table 3:** Haematuria distribution and *COL4A3*/*COL4A4* Gly missense variant features of individuals reported in the 100kGP database.

Characteristic	N haematuria/total individuals (%)		
	*COL4A3*	*COL4A4*	Combined
**(a) All variants (n = 304 individuals)**			
Molecular location			
Exons 1–20	5/45 (11.1%)	7/34 (20.6%)	12/79 (15.2%)
Exons 21 to carboxyl terminus	23/133 (17.3%)	13/92 (14.1%)	36/225 (16.0%)
Collagenous location			
Not adjacent to NC region	27/158 (17.1%)	18/100 (18.0%)	45/258 (17.4%)
Adjacent to NC region	1/20 (5.0%)	2/26 (7.7%)	3/46 (6.5%)
Substituting residue			
Ala/Ser/Cys	5/62 (8.1%)	4/36 (11.1%)	9/98 (9.2%)
Arg/Val/Glu/Asp/Trp	23/116 (19.8%)	16/90 (17.8%)	39/206 (18.9%)
Total	28/178 (15.7%)	20/126 (15.9%)	48/304 (15.8%)
**(b) Excluding NC boundary variants (n = 258 individuals)**			
Local collagenous location			
Central	12/92 (13.0%)	10/71 (14.1%)	22/163 (13.5%)
Amino end	5/28 (17.9%)	6/20 (30.0%)	11/48 (22.9%)
Carboxyl end	10/38 (26.3%)	2/9 (22.2%)	12/47 (25.5%)
Total	27/158 (17.1%)	18/100 (18.0%)	45/258 (17.4%)

NC, non-collagenous.

Location in exons 1–20 was not associated with a difference in risk for haematuria compared with exons 21–53 (p = 0.51). Substitution of a non-collagenous boundary Gly residue was associated with a lower risk for haematuria (p = 0.046), while substitution with a highly destabilising residue was associated with a higher risk (p = 0.018) (Table 4a). However, these features only explained a small proportion of the total variance in haematuria risk (pseudo-R^2^_McFadden_ = 0.040). Excluding all variants affecting a non-collagenous boundary residue, those affecting the amino or carboxyl ends of a local collagenous region were not associated with a difference in risk for haematuria compared with centrally located variants (p = 0.23, p = 0.20 respectively) (Table 4b). Substitution with a highly destabilising residue did not remain significantly associated with a higher risk for haematuria in this subgroup (p = 0.09).

**Table 4:** Logistic regression model of molecular characteristics of *COL4A3* and *COL4A4* Gly missense variants associated with haematuria in the 100kGP database.

	Estimate (SE)	p-value
**(a) All variants (n = 304 individuals)**		
Intercept	− 2.41 (0.47)	** < 0.001**
Location in exons 21 to carboxyl terminus	0.24 (0.37)	0.514
Location adjacent to NC region	− 1.25 (0.62)	**0.046**
Substitution with Arg/Val/Glu/Asp/Trp	0.94 (0.40)	**0.018**
	*Pseudo-R*^2^_*McFadden*_ = *0.040*	
**(b) Excluding NC boundary variants (n = 258 individuals)**		
Intercept	− 2.38 (0.48)	** < 0.001**
Location in exons 21 to carboxy terminus	0.12 (0.39)	0.751
Location at amino end of local collagenous region	0.51 (0.42)	0.226
Location at carboxyl end of local collagenous region	0.55 (0.43)	0.199
Substitution with Arg/Val/Glu/Asp/Trp	0.71 (0.42)	0.092
	*Pseudo-R*^*2*^_*McFadden*_ = *0.033*	

NC, non-collagenous; SE, standard error.

Significant values are in bold.

Two *COL4A3* variants were reported significantly more often in this cohort than any other variant in either gene (Supplemental Fig. 2). These were Gly695Arg (n = 21) and Gly1277Ser (n = 30). Together these accounted for 51/304 (16.8%) of all variants. To ensure that these two variants did not overly influence the results obtained, the logistic regression model was re-evaluated excluding both variants (Supplemental Table 3). Substitution of a non-collagenous boundary residue remained significantly associated with a lower risk for haematuria (p = 0.031), but substitution with a highly destabilising residue now fell just outside the nominal significance level (p = 0.064). The total variance in haematuria explained by the predictor variables remained low (pseudo-R^2^_McFadden_ = 0.043). Interestingly, in the subgroup excluding the non-collagenous boundary variants, location at the carboxyl end of a local collagenous region was now associated with a higher risk for haematuria (p = 0.041).

### Prevalence of *COL4A5* non-collagenous boundary variants in normals

Forty-five unique *COL4A5* Gly missense variants were reported in gnomAD. The proportion of these variants affecting a non-collagenous boundary residue (15/45 = 33.3%) was higher than the expected frequency of 10.1% (p < 0.001). Of interest, the 5 most commonly reported Gly missense variants all affected a non-collagenous boundary residue (Table 5). These 5 variants predominated in single ancestral groups.

**Table 5:** The five most frequently found *COL4A5* Gly missense variants reported in gnomAD.

Nucleotide change	Protein change	Location	Hem	Het	Hom	Total alleles	Most common ethnic groups (n alleles)
2858G>T	Gly953Val	Adjacent to NC region	249	442	7	705	East Asian (n = 552) South Asian (n = 124)
1871G>A	Gly624Asp	Adjacent to NC region	4	12	0	16	European (non-Finnish) (n = 16)
1876G>A	Gly626Ser	Adjacent to NC region	2	3	1	7	European (non-Finnish) (n = 5)
3220G>A	Gly1074Ser	Adjacent to NC region	4	3	0	7	Latino/Admixed American (n = 7)
2882G>T	Gly961Val	Adjacent to NC region	2	3	0	5	Latino/Admixed American (n = 5)

Hem, hemizygotes; Het, heterozygotes; Hom, homozygotes; NC, non-collagenous.

Assuming equal numbers of males and females in gnomAD, missense variants affecting non-collagenous boundary residues were present in 0.6% of the general population (1 in 179 individuals). The majority of these cases were due to a single variant, Gly953Val, which is highly prevalent in East Asian and South Asian populations and currently considered benign^54^. Excluding this variant, missense variants affecting non-collagenous boundary Gly residues were present in 0.05% of the population (1 in 2078 individuals). In contrast, missense variants affecting all other collagenous Gly residues were only present in 0.03% of the general population (1 in 3000 individuals) despite there being nine times as many of these Gly residues in the α5 chain.

## Discussion

Previous studies have demonstrated that the clinical severity of X-linked AS in males is closely associated with truncating, large deletion, splice site, and missense variant types in *COL4A5*^10,24^. This study found that missense variants affecting collagenous Gly residues can be further classified by molecular features that also correlate with severity.

Gly substitutions with highly destabilising residues were associated with an earlier median age at kidney failure. This is consistent with previous studies in other collagen chain genes such as *COL1A1* and *COL1A2*, where Gly substitutions with Arg, Val, Glu or Asp were more likely to result in a lethal phenotype of osteogenesis imperfecta^37^. Similar observations have been seen for *COL4A5* where substitutions with bulkier amino acids lead to an earlier age at kidney failure^35^. Our results are also consistent with the underrepresentation of substitutions with Ala and Ser in pathogenic databases^34,55^, which suggests that they are associated with milder and possibly undiagnosed disease.

Substitutions affecting non-collagenous boundary Gly residues resulted in a delayed age at kidney failure. In general, the non-collagenous interruptions within the collagen type IV chains contribute flexibility and non-pathogenic variants are common in these regions^56^. Gly residues at the boundary of these interruptions may inherit some of this flexibility, which could account for variants’ milder phenotypes. Interestingly, almost all (13/15 = 87%) non-collagenous boundary variants occurred on the amino side of an interruption. Trimerisation occurs in the carboxyl to amino direction, so the side of an interruption that a variant occurs on may affect severity.

Conclusions of the effect of a variant’s molecular location on phenotype severity have been conflicting. Some studies have found that Gly missense variants at the amino end of the collagen IV α5 chain give rise to a milder disease phenotype^38^, while others have found no such relationship^24^. Our study has demonstrated that molecular location was not associated with a difference in age at kidney failure. These results also contradict studies in other collagen genes such as *COL1A1*, that have demonstrated that Gly missense variants occurring at the amino end are more likely to be non-lethal^37^. However, these other collagen types differ from collagen type IV in a number of structural features such as the presence of interruptions and retention of non-collagenous termini, so that a direct comparison may not be appropriate.

In order to deal with the uncertainty associated with the non-collagenous interruptions and the effect of molecular location, each of the 23 local collagenous Gly XY regions was considered as an individual domain with its own amino and carboxyl ends. Surprisingly, analogous to observations in *COL1A1*, variants at the carboxyl end of their local collagenous region were associated with an increased risk of kidney failure. Considering that variants affecting non-collagenous boundary residues have a milder effect than most other Gly variants, the variants affecting the next Gly along were also expected to be milder. This difference may be due to the trimer assembly of the three collagen IV α-chains beginning at the carboxyl end of each chain. Boundary variants on the amino side of an interruption may not be as destructive since they only expand the flexible interruption by one or two residues. However, since the trimers are assembled in the carboxyl to amino direction, a substitution of one or two Gly residues further along may affect the next nucleation-zippering event for trimerization, and result in a more severe phenotype. To our knowledge, this is the first report of such an observation. A previous study of the effect of the distance of a variant to its nearest interruption on age at kidney failure did not demonstrate any relationship^35^.

Substitution with a highly destabilising residue was the only molecular feature associated with an earlier age at diagnosis of hearing loss. The median ages reported here do not predict the age at hearing loss onset, but rather the age at diagnosis. In severe disease, hearing loss onset generally occurs in the first decade, but is often unrecognised and underreported. Affected boys often only undergo audiometry after kidney disease is detected, and usually only when the hearing loss is obvious rather than as a screening test. Nonetheless, these results provide a proof of concept that substitution with a highly destabilising residue has a negative effect on hearing loss phenotype. Variants affecting non-collagenous boundary residues would be expected to also result in a milder hearing loss, but the sample size of this study precluded the demonstration of any differences.

In *COL4A3* and *COL4A4*, substitution of a non-collagenous boundary Gly residue was associated with a lower risk of haematuria, while substitution with a more destabilising residue was associated with a higher risk. This is consistent with our findings in *COL4A5*, where these features were associated with later and earlier ages at kidney failure respectively. However, the proportion of variance in haematuria explained by these features alone was low, and it is likely that other genetic and environmental factors also contributed to haematuria risk. In addition, the control group used here were individuals where haematuria was not formally noted in their medical records. They were not necessarily individuals with a negative urinalysis, and undiagnosed haematuria in this group was probably higher than reported.

A higher proportion of variants affecting non-collagenous boundary Gly residues was observed in gnomAD than expected. The higher frequency of these variants in the general population supports our conclusion that missense variants involving boundary residues are likely to be much milder. Some may even be benign^54^. Additionally, all of the 5 most frequently reported variants affected a boundary residue. One of these (Gly624Asp) has been demonstrated to have originated in Central/East Europe due to a founder effect 750–900 years ago^57^, and other variants have probably also arisen in similar circumstances. We have demonstrated previously that the number of people with a genetic risk for AS in the general population is likely to be higher than currently recognised^4^, and this study’s results suggest that variants affecting non-collagenous boundary residues are major contributors to this largely undiagnosed population.

In this study we have stratified pathogenic *COL4A5* Gly missense variants causing AS into clinically relevant subgroups. We identified molecular features of these variants which are likely to contribute to the clinical severity and disease progression in affected individuals, and provide estimates for the age at kidney failure for each subgroup. However, even within these groups much phenotypic variation still exists, and other genetic factors such as whether a variant affects a ligand binding site^58^ or is located near a proline-rich sequence^59^ may also be important. Environmental factors such as blood pressure control and obesity may also affect the clinical course^60^. Accurate predictions of disease severity and rate of progression are helpful for patients, their clinicians and genetic counsellors, and genetic and clinical data should be considered together when managing patients with AS.

## Supplementary Information

Supplementary Information.
