# Supplementary material for: Genotype–phenotype correlations for COL4A3–COL4A5 variants resulting in Gly substitutions in Alport syndrome
Source: Sci Rep. 2022 Feb 17;12:2722. doi: 10.1038/s41598-022-06525-9 (PMC8854626; doi:10.1038/s41598-022-06525-9)
Supplement: Supplementary file 1 — Supplementary Information. [file 41598_2022_6525_MOESM1_ESM.docx]

**Supplemental Material**

**Table of Contents**

**Supplemental Table 1.** Splicing predictions for all exonic single-nucleotide substitutions occurring within 3 bases of a canonical splice site reported in the LOVD, 100kGP and gnomAD databases.

**Supplemental Table 2.** Classification of all Gly residues in *COL4A3*-*COL4A5*.

**Supplemental Table 3.** Logistic regression model of molecular characteristics of *COL4A3* and *COL4A4* Gly missense variants associated with haematuria in the 100kGP database, excluding two common *COL4A3* variants Gly695Arg and Gly1277Ser.

**Supplemental Figure 1.** Variant inclusion flowcharts for the kidney failure and hearing loss study cohorts.

**Supplemental Figure 2.** *COL4A3* and *COL4A4* Gly missense variants reported in individuals with and without haematuria in the 100kGP database.

**Supplemental Table 1.** Splicing predictions for all exonic single-nucleotide substitutions occurring within 3 bases of a canonical splice site reported in the LOVD, 100kGP and gnomAD databases.

| Gene | Nucleotide change | Expected protein change | Type | MES score (Mutant) | MES score (WT) | MES score change (%) | Predicted to affect splicing |
| --- | --- | --- | --- | --- | --- | --- | --- |
| **a) LOVD** | | | | | | | |
| *COL4A5* | 385G>C | Gly129Arg | Splice acceptor site | 7.81 | 9.46 | -17.44 | Yes |
| *COL4A5* | 386G>A | Gly129Glu | Splice acceptor site | 10.15 | 9.46 | 7.29 | No |
| *COL4A5* | 386G>T | Gly129Val | Splice acceptor site | 10.30 | 9.46 | 8.88 | No |
| *COL4A5* | 611G>A | Gly204Asp | Splice acceptor site | 11.87 | 12.02 | -1.25 | No |
| *COL4A5* | 611G>T | Gly204Val | Splice acceptor site | 12.31 | 12.02 | 2.41 | No |
| *COL4A5* | 646G>A | Gly216Arg | Splice acceptor site | 5.50 | 6.77 | -18.76 | Yes |
| *COL4A5* | 647G>A | Gly216Glu | Splice acceptor site | 6.86 | 6.77 | 1.33 | No |
| *COL4A5* | 647G>T | Gly216Val | Splice acceptor site | 8.22 | 6.77 | 21.42 | No |
| *COL4A5* | 688G>C | Gly230Arg | Splice acceptor site | 7.16 | 8.11 | -11.71 | No |
| *COL4A5* | 688G>T | Gly230Cys | Splice acceptor site | 6.84 | 8.11 | -15.66 | Yes |
| *COL4A5* | 937G>A | Gly313Ser | Splice acceptor site | 5.31 | 7.38 | -28.05 | Yes |
| *COL4A5* | 992G>T | Gly331Val | Splice acceptor site | 5.90 | 4.99 | 18.24 | No |
| *COL4A5* | 1423G>A | Gly475Ser | Splice donor site | -3.12 | 5.13 | -160.82 | Yes |
| *COL4A5* | 1780G>A | Gly594Ser | Splice acceptor site | 11.43 | 12.87 | -11.19 | No |
| *COL4A5* | 1949G>A | Gly650Asp | Splice acceptor site | 10.15 | 10.64 | -4.61 | No |
| *COL4A5* | 2042G>A | Gly681Asp | Splice acceptor site | 10.85 | 12.03 | -9.81 | No |
| *COL4A5* | 2146G>C | Gly716Arg | Splice donor site | 7.69 | 9.79 | -21.45 | Yes |
| *COL4A5* | 2396G>T | Gly799Val | Splice acceptor site | 5.20 | 7.87 | -33.93 | Yes |
| *COL4A5* | 2510G>T | Gly837Val | Splice acceptor site | 2.86 | 5.41 | -47.13 | Yes |
| *COL4A5* | 2678G>A | Gly893Asp | Splice acceptor site | 4.03 | 5.68 | -29.05 | Yes |
| *COL4A5* | 2678G>T | Gly893Val | Splice acceptor site | 3.10 | 5.68 | -45.42 | Yes |
| *COL4A5* | 3017G>T | Gly1006Val | Splice acceptor site | 6.37 | 8.96 | -28.91 | Yes |
| *COL4A5* | 3107G>T | Gly1036Val | Splice acceptor site | 5.97 | 7.58 | -21.24 | Yes |
| *COL4A5* | 3800G>A | Gly1267Asp | Splice acceptor site | 6.50 | 6.90 | -5.80 | No |
| *COL4A5* | 3808G>C | Gly1270Arg | Splice donor site | -3.64 | 6.12 | -159.48 | Yes |
| *COL4A5* | 4087G>A | Gly1363Ser | Splice donor site | 7.61 | 10.77 | -29.34 | Yes |
| *COL4A5* | 4316G>T | Gly1439Val | Splice acceptor site | 2.94 | 5.91 | -50.25 | Yes |
| **b) 100kGP** | | | | | | | |
| *COL4A3* | 145G>C | Gly49Arg | Splice acceptor site | 8.04 | 8.67 | -7.27 | No |
| *COL4A3* | 146G>A | Gly49Glu | Splice acceptor site | 8.77 | 8.67 | 1.15 | No |
| *COL4A3* | 281G>A | Gly94Glu | Splice acceptor site | 11.03 | 10.68 | 3.28 | No |
| *COL4A3* | 443G>T | Gly148Val | Splice acceptor site | 5.70 | 5.45 | 4.59 | No |
| *COL4A3* | 548G>A | Gly183Asp | Splice acceptor site | 8.91 | 8.38 | 6.32 | No |
| *COL4A3* | 611G>T | Gly204Val | Splice acceptor site | 8.98 | 8.19 | 9.65 | No |
| *COL4A3* | 1114G>A | Gly372Ser | Splice donor site | 6.91 | 10.57 | -34.63 | Yes |
| *COL4A3* | 2375G>A | Gly792Glu | Splice acceptor site | 11.81 | 12.46 | -5.22 | No |
| *COL4A3* | 4253G>A | Gly1418Glu | Splice acceptor site | 3.48 | 5.10 | -31.76 | Yes |
| *COL4A4* | 491G>T | Gly164Val | Splice acceptor site | 10.20 | 9.44 | 8.05 | No |
| *COL4A4* | 872G>A | Gly291Glu | Splice acceptor site | 7.78 | 7.10 | 9.58 | No |
| *COL4A4* | 1205G>A | Gly402Asp | Splice acceptor site | 8.89 | 9.03 | -1.55 | No |
| *COL4A4* | 2969G>A | Gly990Asp | Splice acceptor site | 5.66 | 6.67 | -15.14 | Yes |
| *COL4A4* | 3973G>C | Gly1325Arg | Splice donor site | 4.39 | 9.11 | -51.81 | Yes |
| **c) gnomAD** | | | | | | | |
| *COL4A5* | 835G>C | Gly279Arg | Splice acceptor site | 5.77 | 6.04 | -4.47 | No |
| *COL4A5* | 836G>C | Gly279Ala | Splice acceptor site | 5.70 | 6.04 | -5.63 | No |
| *COL4A5* | 2678G>T | Gly893Val | Splice acceptor site | 3.10 | 5.68 | -45.42 | Yes |
| *COL4A5* | 3553G>A | Gly1185Ser | Splice donor site | -4.74 | 4.87 | -197.33 | Yes |

Grey rows indicate variants predicted to affect normal splicing. MES, MaxEntScan; WT, Wild type.

**Supplemental Table 2.** Classification of all Gly residues in *COL4A3*-*COL4A5*.

The three chains are aligned so as to keep all collagenous and non-collagenous regions starting at the same level.

Abbreviations

B Non-collagenous boundary X X of Gly XY (red)

NC Non-collagenous (blue) Y Y of Gly XY (red)

S Adjacent to splice site

* The transcript position of the first base of the codon is listed

| ***COL4A3*** | | |  | ***COL4A4*** | | |  | ***COL4A5*** | | |
| --- | --- | --- | --- | --- | --- | --- | --- | --- | --- | --- |
| Residue | Base* | Notes |  | Residue | Base* | Notes |  | Residue | Base* | Notes |
| **Amino NC domain** | | |  | **Amino NC domain** | | |  | **Amino NC domain** | | |
| Gly30 | c.88 | NC, S |  | Gly22 | c.64 | NC |  | Gly5 | c.13 | NC |
| Gly37 | c.109 | NC |  | Gly38 | c.112 | NC, S |  | Gly11 | c.31 | NC |
|  |  |  |  | Gly40 | c.118 | NC |  | Gly21 | c.61 | NC |
|  |  |  |  | Gly45 | c.133 | NC |  | Gly31 | c.91 | NC |
|  |  |  |  | Gly48 | c.142 | NC |  | Gly35 | c.103 | NC |
|  |  |  |  | Gly49 | c.145 | NC |  |  |  |  |
| Gly43 | c.127 | B |  | Gly62 | c.184 | B |  | Gly42 | c.124 | B |
| Gly46 | c.136 |  |  | Gly65 | c.193 | S |  | Gly45 | c.133 |  |
| Gly49 | c.145 | S |  | Gly68 | c.202 |  |  | Gly48 | c.142 | S |
| Gly52 | c.154 |  |  | Gly71 | c.211 |  |  | Gly51 | c.151 |  |
| Gly55 | c.163 |  |  | Gly74 | c.220 |  |  | Gly54 | c.160 |  |
| Gly58 | c.172 |  |  | Gly77 | c.229 |  |  | Gly57 | c.169 |  |
| Gly61 | c.181 |  |  | Gly80 | c.238 |  |  | Gly60 | c.178 |  |
| Gly64 | c.190 |  |  | Gly83 | c.247 |  |  | Gly63 | c.187 |  |
| Gly67 | c.199 |  |  | Gly86 | c.256 |  |  | Gly66 | c.196 |  |
| Gly70 | c.208 |  |  | Gly89 | c.265 |  |  | Gly69 | c.205 |  |
| Gly73 | c.217 |  |  | Gly92 | c.274 |  |  | Gly72 | c.214 |  |
| Gly76 | c.226 |  |  | Gly95 | c.283 |  |  | Gly75 | c.223 |  |
| Gly79 | c.235 | S |  | Gly98 | c.292 |  |  | Gly78 | c.232 | S |
| Gly82 | c.244 |  |  | Gly101 | c.301 |  |  | Gly81 | c.241 |  |
| Gly85 | c.253 |  |  | Gly104 | c.310 |  |  | Gly84 | c.250 |  |
| Gly88 | c.262 |  |  | Gly107 | c.319 |  |  | Gly87 | c.259 |  |
| Gly91 | c.271 |  |  | Gly110 | c.328 | S |  | Gly90 | c.268 |  |
| Gly94 | c.280 | S |  | Gly113 | c.337 |  |  | Gly93 | c.277 | S |
| Gly97 | c.289 |  |  | Gly116 | c.346 |  |  | Gly96 | c.286 |  |
| Gly100 | c.298 |  |  | Gly119 | c.355 |  |  | Gly99 | c.295 |  |
| Gly103 | c.307 |  |  | Gly122 | c.364 |  |  | Gly102 | c.304 |  |
| Gly106 | c.316 |  |  | Gly125 | c.373 | S |  | Gly105 | c.313 |  |
| Gly109 | c.325 | S |  | Gly128 | c.382 |  |  | Gly108 | c.322 | S |
| Gly112 | c.334 |  |  | Gly131 | c.391 |  |  | Gly111 | c.331 |  |
| Gly115 | c.343 |  |  | Gly134 | c.400 |  |  | Gly114 | c.340 |  |
| Gly118 | c.352 |  |  | Gly137 | c.409 |  |  | Gly117 | c.349 |  |
| Gly121 | c.361 |  |  | Gly140 | c.418 |  |  | Gly120 | c.358 |  |
| Gly124 | c.370 |  |  | Gly143 | c.427 |  |  | Gly123 | c.367 |  |
| Gly127 | c.379 |  |  | Gly146 | c.436 |  |  | Gly126 | c.376 |  |
| Gly130 | c.388 | S |  | Gly149 | c.445 |  |  | Gly129 | c.385 | S |
| Gly133 | c.397 |  |  | Gly152 | c.454 |  |  | Gly132 | c.394 |  |
| Gly136 | c.406 |  |  | Gly153 | c.457 | X |  | Gly135 | c.403 |  |
| Gly139 | c.415 |  |  | Gly155 | c.463 |  |  | Gly138 | c.412 |  |
| Gly142 | c.424 |  |  | Gly158 | c.472 |  |  | Gly141 | c.421 |  |
| Gly145 | c.433 |  |  | Gly160 | c.478 | Y |  | Gly144 | c.430 |  |
| Gly148 | c.442 | S |  | Gly161 | c.481 |  |  | Gly147 | c.439 | S |
| Gly151 | c.451 |  |  | Gly164 | c.490 | S |  | Gly150 | c.448 |  |
| Gly154 | c.460 |  |  | Gly167 | c.499 |  |  | Gly153 | c.457 |  |
| Gly157 | c.469 | B, S |  | Gly170 | c.508 |  |  | Gly156 | c.466 | S |
|  |  |  |  | Gly173 | c.517 | B |  | Gly159 | c.475 | B |
| **NC interruption (I)** | | |  | **NC interruption (I)** | | |  | **NC interruption (I)** | | |
|  |  |  |  | Gly180 | c.538 | NC |  |  |  |  |
| Gly171 | c.511 | B |  | Gly184 | c.550 | B |  | Gly168 | c.502 | B |
| Gly174 | c.520 |  |  | Gly187 | c.559 | S |  | Gly171 | c.511 |  |
| Gly177 | c.529 |  |  | Gly190 | c.568 |  |  | Gly174 | c.520 |  |
| Gly180 | c.538 |  |  | Gly193 | c.577 |  |  | Gly177 | c.529 |  |
| Gly183 | c.547 | S |  | Gly196 | c.586 |  |  | Gly180 | c.538 |  |
| Gly186 | c.556 |  |  | Gly199 | c.595 | S |  | Gly183 | c.547 | S |
| Gly189 | c.565 |  |  | Gly202 | c.604 |  |  | Gly186 | c.556 |  |
| Gly192 | c.574 |  |  | Gly204 | c.610 | Y |  | Gly189 | c.565 |  |
| Gly195 | c.583 |  |  | Gly205 | c.613 |  |  | Gly192 | c.574 |  |
| Gly198 | c.592 |  |  | Gly208 | c.622 |  |  | Gly195 | c.583 |  |
| Gly201 | c.601 |  |  | Gly211 | c.631 |  |  | Gly198 | c.592 |  |
| Gly204 | c.610 | S |  | Gly214 | c.640 |  |  | Gly201 | c.601 |  |
| Gly207 | c.619 |  |  | Gly217 | c.649 |  |  | Gly204 | c.610 | S |
| Gly210 | c.628 |  |  | Gly220 | c.658 | S |  | Gly207 | c.619 |  |
| Gly213 | c.637 |  |  | Gly223 | c.667 |  |  | Gly210 | c.628 |  |
| Gly216 | c.646 | S |  | Gly226 | c.676 |  |  | Gly213 | c.637 |  |
| Gly219 | c.655 | B |  | Gly229 | c.685 |  |  | Gly216 | c.646 | S |
|  |  |  |  | Gly232 | c.694 | S |  | Gly219 | c.655 | B |
|  |  |  |  | Gly235 | c.703 | B |  |  |  |  |
| **NC interruption (II)** | | |  | **NC interruption (II)** | | |  | **NC interruption (II)** | | |
| Gly224 | c.670 | B |  | Gly237 | c.709 | B |  | Gly224 | c.670 | B |
| Gly227 | c.679 |  |  | Gly240 | c.718 |  |  | Gly227 | c.679 |  |
| Gly230 | c.688 | S |  | Gly243 | c.727 |  |  | Gly230 | c.688 | S |
| Gly233 | c.697 |  |  | Gly246 | c.736 | S |  | Gly233 | c.697 |  |
| Gly236 | c.706 |  |  | Gly249 | c.745 |  |  | Gly236 | c.706 |  |
| Gly239 | c.715 |  |  | Gly252 | c.754 |  |  | Gly239 | c.715 |  |
| Gly242 | c.724 | B |  | Gly255 | c.763 | B |  | Gly242 | c.724 | B |
| **NC interruption (III)** | | |  | **NC interruption (III)** | | |  | **NC interruption (III)** | | |
| Gly250 | c.748 | NC |  |  |  |  |  |  |  |  |
| Gly259 | c.775 | B |  | Gly270 | c.808 | B |  | Gly258 | c.772 | B |
| Gly262 | c.784 |  |  | Gly273 | c.817 | S |  | Gly261 | c.781 | S |
| Gly265 | c.793 |  |  | Gly276 | c.826 |  |  | Gly264 | c.790 |  |
| Gly268 | c.802 |  |  | Gly279 | c.835 |  |  | Gly267 | c.799 |  |
| Gly271 | c.811 |  |  | Gly282 | c.844 |  |  | Gly270 | c.808 |  |
| Gly274 | c.820 |  |  | Gly285 | c.853 |  |  | Gly273 | c.817 |  |
| Gly277 | c.829 | S |  | Gly288 | c.862 |  |  | Gly276 | c.826 |  |
| Gly280 | c.838 | B |  | Gly291 | c.871 | S |  | Gly279 | c.835 | S |
|  |  |  |  | Gly294 | c.880 | B |  | Gly282 | c.844 | B |
| **NC interruption (IV)** | | |  | **NC interruption (IV)** | | |  | **NC interruption (IV)** | | |
| Gly284 | c.850 | NC |  |  |  |  |  |  |  |  |
| Gly288 | c.862 | B |  | Gly296 | c.886 | B |  | Gly283 | c.847 | B |
| Gly291 | c.871 |  |  | Gly299 | c.895 |  |  | Gly286 | c.856 |  |
| Gly294 | c.880 |  |  | Gly302 | c.904 |  |  | Gly289 | c.865 |  |
| Gly297 | c.889 | S |  | Gly305 | c.913 |  |  | Gly292 | c.874 |  |
| Gly300 | c.898 |  |  | Gly308 | c.922 |  |  | Gly295 | c.883 |  |
| Gly303 | c.907 |  |  | Gly311 | c.931 | S |  | Gly298 | c.892 | S |
| Gly306 | c.916 |  |  | Gly314 | c.940 |  |  | Gly301 | c.901 |  |
| Gly309 | c.925 |  |  | Gly317 | c.949 |  |  | Gly304 | c.910 |  |
| Gly312 | c.934 | S |  | Gly320 | c.958 |  |  | Gly307 | c.919 |  |
| Gly315 | c.943 |  |  | Gly323 | c.967 |  |  | Gly310 | c.928 |  |
| Gly318 | c.952 |  |  | Gly326 | c.976 | S |  | Gly313 | c.937 | S |
| Gly321 | c.961 |  |  | Gly329 | c.985 |  |  | Gly316 | c.946 |  |
| Gly324 | c.970 |  |  | Gly332 | c.994 |  |  | Gly319 | c.955 |  |
| Gly327 | c.979 |  |  | Gly335 | c.1003 |  |  | Gly322 | c.964 |  |
| Gly330 | c.988 | S |  | Gly338 | c.1012 |  |  | Gly325 | c.973 |  |
| Gly333 | c.997 |  |  | Gly341 | c.1021 |  |  | Gly328 | c.982 |  |
| Gly336 | c.1006 |  |  | Gly344 | c.1030 | S |  | Gly331 | c.991 | S |
| Gly339 | c.1015 |  |  | Gly347 | c.1039 |  |  | Gly334 | c.1000 |  |
| Gly342 | c.1024 | B |  | Gly350 | c.1048 |  |  | Gly337 | c.1009 |  |
|  |  |  |  | Gly353 | c.1057 |  |  | Gly340 | c.1018 |  |
|  |  |  |  | Gly356 | c.1066 | B |  | Gly343 | c.1027 | B |
| **NC interruption (V)** | | |  | **NC interruption (V)** | | |  | **NC interruption (V)** | | |
|  |  |  |  |  |  |  |  | Gly350 | c.1048 | NC |
|  |  |  |  |  |  |  |  | Gly352 | c.1054 | NC |
| Gly354 | c.1060 | B |  | Gly367 | c.1099 | B, S |  | Gly356 | c.1066 | B |
| Gly357 | c.1069 |  |  | Gly370 | c.1108 |  |  | Gly359 | c.1075 |  |
| Gly360 | c.1078 |  |  | Gly373 | c.1117 |  |  | Gly362 | c.1084 |  |
| Gly363 | c.1087 |  |  | Gly376 | c.1126 |  |  | Gly365 | c.1093 |  |
| Gly366 | c.1096 |  |  | Gly379 | c.1135 |  |  | Gly368 | c.1102 |  |
| Gly369 | c.1105 |  |  | Gly382 | c.1144 |  |  | Gly371 | c.1111 |  |
| Gly372 | c.1114 | S |  | Gly385 | c.1153 |  |  | Gly374 | c.1120 |  |
| Gly375 | c.1123 |  |  | Gly388 | c.1162 |  |  | Gly377 | c.1129 |  |
| Gly378 | c.1132 |  |  | Gly391 | c.1171 |  |  | Gly380 | c.1138 |  |
| Gly381 | c.1141 |  |  | Gly394 | c.1180 |  |  | Gly383 | c.1147 |  |
| Gly384 | c.1150 | B, S |  | Gly397 | c.1189 | B |  | Gly386 | c.1156 |  |
|  |  |  |  |  |  |  |  | Gly389 | c.1165 | B, S |
| **NC interruption (VI)** | | |  | **NC interruption (VI)** | | |  | **NC interruption (VI)** | | |
| Gly389 | c.1165 | B |  | Gly402 | c.1204 | B, S |  | Gly394 | c.1180 | B |
| Gly392 | c.1174 |  |  | Gly405 | c.1213 |  |  | Gly397 | c.1189 |  |
| Gly395 | c.1183 |  |  | Gly408 | c.1222 |  |  | Gly400 | c.1198 |  |
| Gly398 | c.1192 |  |  | Gly411 | c.1231 |  |  | Gly403 | c.1207 |  |
| Gly401 | c.1201 |  |  | Gly414 | c.1240 |  |  | Gly406 | c.1216 |  |
| Gly404 | c.1210 |  |  | Gly417 | c.1249 |  |  | Gly409 | c.1225 |  |
| Gly407 | c.1219 |  |  | Gly420 | c.1258 |  |  | Gly412 | c.1234 |  |
| Gly410 | c.1228 | B |  | Gly423 | c.1267 |  |  | Gly415 | c.1243 | B |
|  |  |  |  | Gly426 | c.1276 | B |  |  |  |  |
| **NC interruption (VII)** | | |  | **NC interruption (VII)** | | |  | **NC interruption (VII)** | | |
| Gly415 | c.1243 | B |  | Gly433 | c.1297 | B |  | Gly420 | c.1258 | B |
| Gly418 | c.1252 |  |  | Gly436 | c.1306 |  |  | Gly423 | c.1267 |  |
| Gly421 | c.1261 |  |  | Gly439 | c.1315 |  |  | Gly426 | c.1276 |  |
| Gly424 | c.1270 |  |  | Gly442 | c.1324 |  |  | Gly429 | c.1285 |  |
| Gly427 | c.1279 |  |  | Gly445 | c.1333 |  |  | Gly432 | c.1294 |  |
| Gly430 | c.1288 |  |  | Gly448 | c.1342 |  |  | Gly435 | c.1303 |  |
| Gly433 | c.1297 |  |  | Gly451 | c.1351 |  |  | Gly438 | c.1312 |  |
| Gly436 | c.1306 |  |  | Gly454 | c.1360 | B |  | Gly441 | c.1321 | B |
| Gly439 | c.1315 | B, S |  |  |  |  |  |  |  |  |
| **NC interruption (VIII)** | | |  | **NC interruption (VIII)** | | |  | **NC interruption (VIII)** | | |
| Gly446 | c.1336 | B |  | Gly463 | c.1387 | B |  | Gly454 | c.1360 | B |
| Gly449 | c.1345 |  |  | Gly466 | c.1396 |  |  | Gly457 | c.1369 |  |
| Gly452 | c.1354 |  |  | Gly469 | c.1405 |  |  | Gly460 | c.1378 |  |
| Gly455 | c.1363 |  |  | Gly472 | c.1414 |  |  | Gly463 | c.1387 |  |
| Gly458 | c.1372 |  |  | Gly475 | c.1423 |  |  | Gly466 | c.1396 |  |
| Gly461 | c.1381 |  |  | Gly478 | c.1432 |  |  | Gly469 | c.1405 |  |
| Gly464 | c.1390 |  |  | Gly479 | c.1435 | X |  | Gly472 | c.1414 |  |
| Gly467 | c.1399 |  |  | Gly481 | c.1441 |  |  | Gly475 | c.1423 | S |
| Gly470 | c.1408 | S |  | Gly484 | c.1450 |  |  | Gly478 | c.1432 | B |
| Gly473 | c.1417 | B |  | Gly487 | c.1459 | S |  |  |  |  |
|  |  |  |  | Gly490 | c.1468 | B |  |  |  |  |
| **NC interruption (IX)** | | |  | **NC interruption (IX)** | | |  | **NC interruption (IX)** | | |
|  |  |  |  |  |  |  |  | Gly486 | c.1456 | NC |
| Gly484 | c.1450 | B |  | Gly497 | c.1489 | B |  | Gly488 | c.1462 | B |
| Gly487 | c.1459 |  |  | Gly500 | c.1498 |  |  | Gly491 | c.1471 |  |
| Gly490 | c.1468 |  |  | Gly503 | c.1507 |  |  | Gly494 | c.1480 |  |
| Gly493 | c.1477 |  |  | Gly506 | c.1516 |  |  | Gly497 | c.1489 |  |
| Gly496 | c.1486 |  |  | Gly509 | c.1525 |  |  | Gly500 | c.1498 |  |
| Gly499 | c.1495 |  |  | Gly512 | c.1534 |  |  | Gly503 | c.1507 |  |
| Gly502 | c.1504 | S |  | Gly515 | c.1543 |  |  | Gly506 | c.1516 | S |
| Gly505 | c.1513 |  |  | Gly518 | c.1552 |  |  | Gly509 | c.1525 |  |
| Gly508 | c.1522 |  |  | Gly521 | c.1561 |  |  | Gly512 | c.1534 |  |
| Gly511 | c.1531 |  |  | Gly524 | c.1570 |  |  | Gly515 | c.1543 |  |
| Gly514 | c.1540 |  |  | Gly527 | c.1579 |  |  | Gly518 | c.1552 |  |
| Gly517 | c.1549 |  |  | Gly530 | c.1588 |  |  | Gly521 | c.1561 |  |
| Gly520 | c.1558 |  |  | Gly533 | c.1597 |  |  | Gly524 | c.1570 |  |
| Gly523 | c.1567 |  |  | Gly536 | c.1606 |  |  | Gly527 | c.1579 |  |
| Gly526 | c.1576 | S |  | Gly539 | c.1615 |  |  | Gly530 | c.1588 | S |
| Gly529 | c.1585 |  |  | Gly542 | c.1624 | S |  | Gly533 | c.1597 |  |
| Gly532 | c.1594 |  |  | Gly545 | c.1633 |  |  | Gly536 | c.1606 |  |
| Gly535 | c.1603 |  |  | Gly548 | c.1642 |  |  | Gly539 | c.1615 |  |
| Gly538 | c.1612 |  |  | Gly551 | c.1651 |  |  | Gly542 | c.1624 |  |
| Gly541 | c.1621 | B |  | Gly554 | c.1660 |  |  | Gly545 | c.1633 |  |
|  |  |  |  | Gly557 | c.1669 | B |  | Gly548 | c.1642 | B |
| **NC interruption (X)** | | |  | **NC interruption (X)** | | |  | **NC interruption (X)** | | |
| Gly548 | c.1642 | B |  | Gly566 | c.1696 | B, S |  | Gly555 | c.1663 | B |
| Gly551 | c.1651 |  |  | Gly569 | c.1705 |  |  | Gly558 | c.1672 |  |
| Gly554 | c.1660 |  |  | Gly572 | c.1714 |  |  | Gly561 | c.1681 |  |
| Gly557 | c.1669 |  |  | Gly575 | c.1723 |  |  | Gly564 | c.1690 |  |
| Gly560 | c.1678 |  |  | Gly578 | c.1732 |  |  | Gly567 | c.1699 |  |
| Gly563 | c.1687 |  |  | Gly581 | c.1741 |  |  | Gly570 | c.1708 |  |
| Gly566 | c.1696 |  |  | Gly584 | c.1750 |  |  | Gly573 | c.1717 |  |
| Gly569 | c.1705 |  |  | Gly587 | c.1759 |  |  | Gly576 | c.1726 |  |
| Gly572 | c.1714 |  |  | Gly590 | c.1768 |  |  | Gly579 | c.1735 |  |
| Gly575 | c.1723 |  |  | Gly593 | c.1777 |  |  | Gly582 | c.1744 |  |
| Gly578 | c.1732 |  |  | Gly596 | c.1786 |  |  | Gly585 | c.1753 |  |
| Gly581 | c.1741 |  |  | Gly599 | c.1795 |  |  | Gly588 | c.1762 |  |
| Gly584 | c.1750 | B |  | Gly602 | c.1804 | B, S |  | Gly591 | c.1771 |  |
|  |  |  |  |  |  |  |  | Gly594 | c.1780 | B, S |
| **NC interruption (XI)** | | |  | **NC interruption (XI)** | | |  | **NC interruption (XI)** | | |
|  |  |  |  |  |  |  |  | Gly595 | c.1783 | NC |
| Gly590 | c.1768 | B |  | Gly610 | c.1828 | B |  | Gly600 | c.1798 | B |
| Gly593 | c.1777 |  |  | Gly611 | c.1831 | X |  | Gly603 | c.1807 |  |
| Gly596 | c.1786 |  |  | Gly613 | c.1837 |  |  | Gly606 | c.1816 |  |
| Gly599 | c.1795 |  |  | Gly616 | c.1846 |  |  | Gly609 | c.1825 |  |
| Gly602 | c.1804 |  |  | Gly619 | c.1855 |  |  | Gly612 | c.1834 |  |
| Gly605 | c.1813 |  |  | Gly622 | c.1864 |  |  | Gly615 | c.1843 |  |
| Gly608 | c.1822 |  |  | Gly625 | c.1873 |  |  | Gly618 | c.1852 |  |
| Gly611 | c.1831 |  |  | Gly628 | c.1882 |  |  | Gly621 | c.1861 |  |
| Gly614 | c.1840 |  |  | Gly631 | c.1891 | B |  | Gly624 | c.1870 | B |
| Gly617 | c.1849 | B |  |  |  |  |  |  |  |  |
| **NC interruption (XII)** | | |  | **NC interruption (XII)** | | |  | **NC interruption (XII)** | | |
| Gly619 | c.1855 | B |  | Gly633 | c.1897 | B |  | Gly626 | c.1876 | B |
| Gly622 | c.1864 |  |  | Gly636 | c.1906 |  |  | Gly629 | c.1885 |  |
| Gly625 | c.1873 |  |  | Gly639 | c.1915 |  |  | Gly632 | c.1894 |  |
| Gly628 | c.1882 |  |  | Gly642 | c.1924 |  |  | Gly635 | c.1903 |  |
| Gly631 | c.1891 |  |  | Gly645 | c.1933 |  |  | Gly638 | c.1912 |  |
| Gly634 | c.1900 |  |  | Gly648 | c.1942 |  |  | Gly641 | c.1921 |  |
| Gly637 | c.1909 |  |  | Gly651 | c.1951 |  |  | Gly644 | c.1930 |  |
| Gly640 | c.1918 |  |  | Gly654 | c.1960 |  |  | Gly647 | c.1939 |  |
| Gly643 | c.1927 | S |  | Gly657 | c.1969 |  |  | Gly650 | c.1948 | S |
| Gly646 | c.1936 | B |  | Gly660 | c.1978 |  |  | Gly653 | c.1957 |  |
|  |  |  |  | Gly663 | c.1987 | B, S |  | Gly656 | c.1966 | B |
| **NC interruption (XIII)** | | |  | **NC interruption (XIII)** | | |  | **NC interruption (XIII)** | | |
| Gly656 | c.1966 | B |  | Gly674 | c.2020 | B |  | Gly663 | c.1987 | B |
| Gly659 | c.1975 |  |  | Gly677 | c.2029 |  |  | Gly666 | c.1996 |  |
| Gly662 | c.1984 |  |  | Gly680 | c.2038 |  |  | Gly669 | c.2005 |  |
| Gly665 | c.1993 |  |  | Gly683 | c.2047 |  |  | Gly672 | c.2014 |  |
| Gly668 | c.2002 |  |  | Gly686 | c.2056 | S |  | Gly675 | c.2023 |  |
| Gly671 | c.2011 |  |  | Gly689 | c.2065 |  |  | Gly678 | c.2032 |  |
| Gly674 | c.2020 | S |  | Gly692 | c.2074 |  |  | Gly681 | c.2041 | S |
| Gly677 | c.2029 |  |  | Gly695 | c.2083 |  |  | Gly684 | c.2050 |  |
| Gly680 | c.2038 |  |  | Gly698 | c.2092 |  |  | Gly687 | c.2059 |  |
| Gly683 | c.2047 |  |  | Gly701 | c.2101 |  |  | Gly690 | c.2068 |  |
| Gly686 | c.2056 |  |  | Gly704 | c.2110 |  |  | Gly693 | c.2077 |  |
| Gly689 | c.2065 |  |  | Gly707 | c.2119 |  |  | Gly696 | c.2086 |  |
| Gly692 | c.2074 |  |  | Gly710 | c.2128 |  |  | Gly699 | c.2095 |  |
| Gly695 | c.2083 |  |  | Gly713 | c.2137 | B |  | Gly702 | c.2104 |  |
| Gly698 | c.2092 | B |  |  |  |  |  | Gly705 | c.2113 | B |
| **NC interruption (XIV)** | | |  | **NC interruption (XIV)** | | |  | **NC interruption (XIV)** | | |
| Gly700 | c.2098 | B |  | Gly719 | c.2155 | B |  | Gly707 | c.2119 | B |
| Gly703 | c.2107 |  |  | Gly722 | c.2164 | S |  | Gly710 | c.2128 |  |
| Gly706 | c.2116 |  |  | Gly725 | c.2173 |  |  | Gly713 | c.2137 |  |
| Gly709 | c.2125 | S |  | Gly728 | c.2182 |  |  | Gly716 | c.2146 | S |
| Gly712 | c.2134 |  |  | Gly731 | c.2191 |  |  | Gly719 | c.2155 |  |
| Gly715 | c.2143 |  |  | Gly733 | c.2197 | Y |  | Gly722 | c.2164 |  |
| Gly718 | c.2152 |  |  | Gly734 | c.2200 |  |  | Gly725 | c.2173 |  |
| Gly721 | c.2161 |  |  | Gly737 | c.2209 | B |  | Gly728 | c.2182 |  |
| Gly724 | c.2170 |  |  | **NC interruption (XV)** | | |  | Gly731 | c.2191 |  |
| Gly727 | c.2179 |  |  | Gly742 | c.2224 | B |  | Gly734 | c.2200 |  |
| Gly730 | c.2188 |  |  | Gly745 | c.2233 |  |  | Gly737 | c.2209 |  |
| Gly733 | c.2197 |  |  | Gly748 | c.2242 |  |  | Gly740 | c.2218 |  |
| Gly736 | c.2206 |  |  | Gly751 | c.2251 |  |  | Gly743 | c.2227 |  |
| Gly739 | c.2215 |  |  | Gly754 | c.2260 |  |  | Gly746 | c.2236 |  |
| Gly742 | c.2224 | B, S |  | Gly757 | c.2269 |  |  | Gly749 | c.2245 | S |
|  |  |  |  | Gly760 | c.2278 | B |  | Gly752 | c.2254 | B |
| **NC interruption (XV)** | | |  | **NC interruption (XVI)** | | |  | **NC interruption (XV)** | | |
| Gly750 | c.2248 | B |  | Gly765 | c.2293 | B |  | Gly757 | c.2269 | B |
| Gly751 | c.2251 | X |  | Gly768 | c.2302 |  |  | Gly760 | c.2278 |  |
| Gly753 | c.2257 |  |  | Gly771 | c.2311 |  |  | Gly763 | c.2287 |  |
| Gly756 | c.2266 |  |  | Gly774 | c.2320 |  |  | Gly766 | c.2296 |  |
| Gly759 | c.2275 |  |  | Gly777 | c.2329 |  |  | Gly769 | c.2305 |  |
| Gly762 | c.2284 |  |  | Gly780 | c.2338 |  |  | Gly772 | c.2314 |  |
| Gly765 | c.2293 |  |  | Gly783 | c.2347 |  |  | Gly775 | c.2323 |  |
| Gly768 | c.2302 |  |  | Gly786 | c.2356 |  |  | Gly778 | c.2332 |  |
| Gly771 | c.2311 |  |  | Gly789 | c.2365 |  |  | Gly781 | c.2341 |  |
| Gly774 | c.2320 |  |  | Gly792 | c.2374 |  |  | Gly784 | c.2350 |  |
| Gly777 | c.2329 |  |  | Gly795 | c.2383 | S |  | Gly787 | c.2359 |  |
| Gly780 | c.2338 |  |  | Gly798 | c.2392 |  |  | Gly790 | c.2368 |  |
| Gly783 | c.2347 |  |  | Gly801 | c.2401 |  |  | Gly793 | c.2377 |  |
| Gly786 | c.2356 |  |  | Gly804 | c.2410 |  |  | Gly796 | c.2386 |  |
| Gly789 | c.2365 |  |  | Gly807 | c.2419 |  |  | Gly799 | c.2395 | S |
| Gly792 | c.2374 | S |  | Gly810 | c.2428 |  |  | Gly802 | c.2404 |  |
| Gly795 | c.2383 |  |  | Gly813 | c.2437 |  |  | Gly805 | c.2413 |  |
| Gly798 | c.2392 |  |  | Gly816 | c.2446 |  |  | Gly808 | c.2422 |  |
| Gly801 | c.2401 |  |  | Gly819 | c.2455 |  |  | Gly811 | c.2431 |  |
| Gly804 | c.2410 |  |  | Gly822 | c.2464 |  |  | Gly814 | c.2440 |  |
| Gly807 | c.2419 | B |  | Gly825 | c.2473 | B |  | Gly817 | c.2449 | B |
| **NC interruption (XVI)** | | |  | **NC interruption (XVII)** | | |  | **NC interruption (XVI)** | | |
| Gly812 | c.2434 | B |  | Gly831 | c.2491 | B |  | Gly819 | c.2455 | B |
| Gly815 | c.2443 |  |  | Gly834 | c.2500 |  |  | Gly822 | c.2464 |  |
| Gly818 | c.2452 |  |  | Gly837 | c.2509 |  |  | Gly825 | c.2473 |  |
| Gly821 | c.2461 |  |  | Gly840 | c.2518 |  |  | Gly828 | c.2482 |  |
| Gly824 | c.2470 |  |  | Gly843 | c.2527 |  |  | Gly831 | c.2491 |  |
| Gly827 | c.2479 |  |  | Gly846 | c.2536 |  |  | Gly834 | c.2500 |  |
| Gly830 | c.2488 | S |  | Gly849 | c.2545 | S |  | Gly837 | c.2509 | S |
| Gly833 | c.2497 |  |  | Gly852 | c.2554 |  |  | Gly840 | c.2518 |  |
| Gly836 | c.2506 |  |  | Gly853 | c.2557 | X |  | Gly843 | c.2527 |  |
| Gly839 | c.2515 |  |  | Gly855 | c.2563 |  |  | Gly846 | c.2536 |  |
| Gly842 | c.2524 |  |  | Gly858 | c.2572 |  |  | Gly849 | c.2545 |  |
| Gly845 | c.2533 | B |  | Gly861 | c.2581 |  |  | Gly852 | c.2554 | B |
| **NC interruption (XVII)** | | |  | Gly864 | c.2590 |  |  | **NC interruption (XVII)** | | |
| Gly850 | c.2548 | B |  | Gly867 | c.2599 |  |  | Gly857 | c.2569 | B |
| Gly853 | c.2557 |  |  | Gly870 | c.2608 |  |  | Gly860 | c.2578 |  |
| Gly856 | c.2566 |  |  | Gly873 | c.2617 |  |  | Gly863 | c.2587 |  |
| Gly859 | c.2575 |  |  | Gly876 | c.2626 |  |  | Gly866 | c.2596 |  |
| Gly862 | c.2584 |  |  | Gly879 | c.2635 |  |  | Gly869 | c.2605 |  |
| Gly865 | c.2593 |  |  | Gly882 | c.2644 |  |  | Gly872 | c.2614 |  |
| Gly868 | c.2602 |  |  | Gly885 | c.2653 |  |  | Gly875 | c.2623 |  |
| Gly871 | c.2611 |  |  | Gly888 | c.2662 |  |  | Gly878 | c.2632 |  |
| Gly874 | c.2620 |  |  | Gly891 | c.2671 |  |  | Gly881 | c.2641 |  |
| Gly877 | c.2629 |  |  | Gly894 | c.2680 |  |  | Gly884 | c.2650 |  |
| Gly880 | c.2638 |  |  | Gly897 | c.2689 |  |  | Gly887 | c.2659 |  |
| Gly883 | c.2647 |  |  | Gly900 | c.2698 |  |  | Gly890 | c.2668 |  |
| Gly886 | c.2656 | S |  | Gly903 | c.2707 |  |  | Gly893 | c.2677 | S |
| Gly889 | c.2665 |  |  | Gly906 | c.2716 | S |  | Gly896 | c.2686 |  |
| Gly892 | c.2674 |  |  | Gly909 | c.2725 |  |  | Gly899 | c.2695 |  |
| Gly895 | c.2683 |  |  | Gly912 | c.2734 |  |  | Gly902 | c.2704 |  |
| Gly898 | c.2692 |  |  | Gly915 | c.2743 |  |  | Gly905 | c.2713 |  |
| Gly901 | c.2701 |  |  | Gly918 | c.2752 |  |  | Gly908 | c.2722 |  |
| Gly904 | c.2710 |  |  | Gly921 | c.2761 |  |  | Gly911 | c.2731 |  |
| Gly907 | c.2719 |  |  | Gly924 | c.2770 |  |  | Gly914 | c.2740 |  |
| Gly910 | c.2728 |  |  | Gly927 | c.2779 |  |  | Gly917 | c.2749 |  |
| Gly913 | c.2737 |  |  | Gly930 | c.2788 |  |  | Gly920 | c.2758 |  |
| Gly916 | c.2746 | S |  | Gly933 | c.2797 |  |  | Gly923 | c.2767 | S |
| Gly919 | c.2755 |  |  | Gly936 | c.2806 |  |  | Gly926 | c.2776 |  |
| Gly922 | c.2764 |  |  | Gly939 | c.2815 |  |  | Gly929 | c.2785 |  |
| Gly925 | c.2773 |  |  | Gly942 | c.2824 |  |  | Gly932 | c.2794 |  |
| Gly928 | c.2782 |  |  | Gly945 | c.2833 |  |  | Gly935 | c.2803 |  |
| Gly931 | c.2791 |  |  | Gly948 | c.2842 |  |  | Gly938 | c.2812 |  |
| Gly934 | c.2800 |  |  | Gly951 | c.2851 |  |  | Gly941 | c.2821 |  |
| Gly937 | c.2809 |  |  | Gly954 | c.2860 | S |  | Gly944 | c.2830 |  |
| Gly940 | c.2818 |  |  | Gly957 | c.2869 |  |  | Gly947 | c.2839 |  |
| Gly943 | c.2827 | B |  | Gly960 | c.2878 |  |  | Gly950 | c.2848 |  |
|  |  |  |  | Gly963 | c.2887 | B |  | Gly953 | c.2857 | B |
| **NC interruption (XVIII)** | | |  | **NC interruption (XVIII)** | | |  | **NC interruption (XVIII)** | | |
| Gly952 | c.2854 | B |  | Gly972 | c.2914 | B |  | Gly961 | c.2881 | B |
| Gly955 | c.2863 |  |  | Gly975 | c.2923 |  |  | Gly964 | c.2890 |  |
| Gly958 | c.2872 |  |  | Gly978 | c.2932 |  |  | Gly967 | c.2899 |  |
| Gly961 | c.2881 | S |  | Gly981 | c.2941 |  |  | Gly970 | c.2908 |  |
| Gly964 | c.2890 |  |  | Gly984 | c.2950 |  |  | Gly973 | c.2917 | S |
| Gly967 | c.2899 |  |  | Gly987 | c.2959 |  |  | Gly976 | c.2926 |  |
| Gly970 | c.2908 |  |  | Gly990 | c.2968 | S |  | Gly979 | c.2935 |  |
| Gly973 | c.2917 |  |  | Gly993 | c.2977 |  |  | Gly982 | c.2944 |  |
| Gly976 | c.2926 |  |  | Gly996 | c.2986 |  |  | Gly985 | c.2953 |  |
| Gly979 | c.2935 |  |  | Gly999 | c.2995 |  |  | Gly988 | c.2962 |  |
| Gly982 | c.2944 |  |  | Gly1002 | c.3004 |  |  | Gly991 | c.2971 |  |
| Gly985 | c.2953 |  |  | Gly1005 | c.3013 |  |  | Gly994 | c.2980 |  |
| Gly988 | c.2962 |  |  | Gly1008 | c.3022 |  |  | Gly997 | c.2989 |  |
| Gly991 | c.2971 |  |  | Gly1011 | c.3031 | B |  | Gly1000 | c.2998 |  |
| Gly994 | c.2980 | S |  | **NC interruption (XIX)** | | |  | Gly1003 | c.3007 |  |
| Gly997 | c.2989 |  |  | Gly1015 | c.3043 | B |  | Gly1006 | c.3016 | S |
| Gly1000 | c.2998 |  |  | Gly1018 | c.3052 |  |  | Gly1009 | c.3025 |  |
| Gly1003 | c.3007 |  |  | Gly1021 | c.3061 |  |  | Gly1012 | c.3034 |  |
| Gly1006 | c.3016 |  |  | Gly1024 | c.3070 |  |  | Gly1015 | c.3043 |  |
| Gly1009 | c.3025 |  |  | Gly1027 | c.3079 |  |  | Gly1018 | c.3052 |  |
| Gly1012 | c.3034 |  |  | Gly1030 | c.3088 |  |  | Gly1021 | c.3061 |  |
| Gly1015 | c.3043 |  |  | Gly1033 | c.3097 |  |  | Gly1024 | c.3070 |  |
| Gly1018 | c.3052 |  |  | Gly1036 | c.3106 |  |  | Gly1027 | c.3079 |  |
| Gly1021 | c.3061 |  |  | Gly1039 | c.3115 |  |  | Gly1030 | c.3088 |  |
| Gly1024 | c.3070 | S |  | Gly1042 | c.3124 |  |  | Gly1033 | c.3097 |  |
| Gly1027 | c.3079 |  |  | Gly1045 | c.3133 |  |  | Gly1036 | c.3106 | S |
| Gly1030 | c.3088 |  |  | Gly1048 | c.3142 |  |  | Gly1039 | c.3115 |  |
| Gly1033 | c.3097 |  |  | Gly1051 | c.3151 | S |  | Gly1042 | c.3124 |  |
| Gly1036 | c.3106 |  |  | Gly1054 | c.3160 |  |  | Gly1045 | c.3133 |  |
| Gly1039 | c.3115 |  |  | Gly1057 | c.3169 |  |  | Gly1048 | c.3142 |  |
| Gly1042 | c.3124 |  |  | Gly1060 | c.3178 |  |  | Gly1051 | c.3151 |  |
| Gly1045 | c.3133 |  |  | Gly1063 | c.3187 |  |  | Gly1054 | c.3160 |  |
| Gly1048 | c.3142 |  |  | Gly1066 | c.3196 |  |  | Gly1057 | c.3169 |  |
| Gly1051 | c.3151 |  |  | Gly1069 | c.3205 |  |  | Gly1060 | c.3178 |  |
| Gly1054 | c.3160 |  |  | Gly1072 | c.3214 | S |  | Gly1063 | c.3187 |  |
| Gly1057 | c.3169 | B |  | Gly1075 | c.3223 | B |  | Gly1066 | c.3196 |  |
|  |  |  |  |  |  |  |  | Gly1069 | c.3205 | B |
| **NC interruption (XIX)** | | |  | **NC interruption (XX)** | | |  | **NC interruption (XIX)** | | |
| Gly1061 | c.3181 | NC |  |  |  |  |  |  |  |  |
| Gly1065 | c.3193 | B |  | Gly1082 | c.3244 | B |  | Gly1074 | c.3220 | B |
| Gly1068 | c.3202 |  |  | Gly1085 | c.3253 |  |  | Gly1077 | c.3229 |  |
| Gly1071 | c.3211 | S |  | Gly1088 | c.3262 |  |  | Gly1080 | c.3238 |  |
| Gly1074 | c.3220 |  |  | Gly1091 | c.3271 |  |  | Gly1083 | c.3247 | S |
| Gly1077 | c.3229 |  |  | Gly1094 | c.3280 |  |  | Gly1086 | c.3256 |  |
| Gly1080 | c.3238 |  |  | Gly1097 | c.3289 | S |  | Gly1089 | c.3265 |  |
| Gly1083 | c.3247 |  |  | Gly1100 | c.3298 |  |  | Gly1092 | c.3274 |  |
| Gly1086 | c.3256 |  |  | Gly1103 | c.3307 |  |  | Gly1095 | c.3283 |  |
| Gly1089 | c.3265 |  |  | Gly1106 | c.3316 |  |  | Gly1098 | c.3292 |  |
| Gly1092 | c.3274 |  |  | Gly1109 | c.3325 |  |  | Gly1101 | c.3301 |  |
| Gly1095 | c.3283 |  |  | Gly1112 | c.3334 |  |  | Gly1104 | c.3310 |  |
| Gly1098 | c.3292 |  |  | Gly1115 | c.3343 |  |  | Gly1107 | c.3319 |  |
| Gly1101 | c.3301 |  |  | Gly1118 | c.3352 |  |  | Gly1110 | c.3328 |  |
| Gly1104 | c.3310 |  |  | Gly1121 | c.3361 |  |  | Gly1113 | c.3337 |  |
| Gly1107 | c.3319 |  |  | Gly1124 | c.3370 |  |  | Gly1116 | c.3346 |  |
| Gly1110 | c.3328 |  |  | Gly1127 | c.3379 |  |  | Gly1119 | c.3355 |  |
| Gly1113 | c.3337 | S |  | Gly1130 | c.3388 |  |  | Gly1122 | c.3364 |  |
| Gly1116 | c.3346 |  |  | Gly1133 | c.3397 | S |  | Gly1125 | c.3373 | S |
| Gly1119 | c.3355 |  |  | Gly1136 | c.3406 |  |  | Gly1128 | c.3382 |  |
| Gly1122 | c.3364 |  |  | Gly1139 | c.3415 |  |  | Gly1131 | c.3391 |  |
| Gly1125 | c.3373 |  |  | Gly1142 | c.3424 |  |  | Gly1134 | c.3400 |  |
| Gly1128 | c.3382 |  |  | Gly1145 | c.3433 |  |  | Gly1137 | c.3409 |  |
| Gly1131 | c.3391 |  |  | Gly1148 | c.3442 |  |  | Gly1140 | c.3418 |  |
| Gly1134 | c.3400 |  |  | Gly1151 | c.3451 |  |  | Gly1143 | c.3427 |  |
| Gly1137 | c.3409 |  |  | Gly1154 | c.3460 |  |  | Gly1146 | c.3436 |  |
| Gly1140 | c.3418 | S |  | Gly1157 | c.3469 |  |  | Gly1149 | c.3445 |  |
| Gly1143 | c.3427 |  |  | Gly1160 | c.3478 |  |  | Gly1152 | c.3454 | S |
| Gly1146 | c.3436 |  |  | Gly1163 | c.3487 |  |  | Gly1153 | c.3457 | X |
| Gly1149 | c.3445 |  |  | Gly1166 | c.3496 |  |  | Gly1154 | c.3460 | Y |
| Gly1152 | c.3454 |  |  | Gly1169 | c.3505 | S |  | Gly1155 | c.3463 |  |
| Gly1155 | c.3463 |  |  | Gly1172 | c.3514 |  |  | Gly1158 | c.3472 |  |
| Gly1158 | c.3472 |  |  | Gly1175 | c.3523 |  |  | Gly1161 | c.3481 |  |
| Gly1161 | c.3481 |  |  | Gly1178 | c.3532 |  |  | Gly1164 | c.3490 |  |
| Gly1164 | c.3490 |  |  | Gly1181 | c.3541 |  |  | Gly1167 | c.3499 |  |
| Gly1167 | c.3499 |  |  | Gly1184 | c.3550 |  |  | Gly1170 | c.3508 |  |
| Gly1170 | c.3508 |  |  | Gly1187 | c.3559 |  |  | Gly1173 | c.3517 |  |
| Gly1173 | c.3517 | B, S |  | Gly1190 | c.3568 |  |  | Gly1176 | c.3526 |  |
|  |  |  |  | Gly1193 | c.3577 | B, S |  | Gly1179 | c.3535 |  |
|  |  |  |  |  |  |  |  | Gly1182 | c.3544 |  |
|  |  |  |  |  |  |  |  | Gly1185 | c.3553 | S |
|  |  |  |  |  |  |  |  | Gly1188 | c.3562 | B |
| **NC interruption (XX)** | | |  | **NC interruption (XXI)** | | |  | **NC interruption (XX)** | | |
| Gly1180 | c.3538 | B |  | Gly1198 | c.3592 | B |  | Gly1190 | c.3568 | B |
| Gly1183 | c.3547 |  |  | Gly1201 | c.3601 |  |  | Gly1193 | c.3577 |  |
| Gly1186 | c.3556 |  |  | Gly1204 | c.3610 |  |  | Gly1196 | c.3586 |  |
| Gly1189 | c.3565 | S |  | Gly1207 | c.3619 |  |  | Gly1199 | c.3595 |  |
| Gly1192 | c.3574 |  |  | Gly1210 | c.3628 |  |  | Gly1202 | c.3604 | S |
| Gly1195 | c.3583 |  |  | Gly1213 | c.3637 |  |  | Gly1205 | c.3613 |  |
| Gly1198 | c.3592 |  |  | Gly1216 | c.3646 |  |  | Gly1207 | c.3619 | Y |
| Gly1201 | c.3601 |  |  | Gly1219 | c.3655 | B |  | Gly1208 | c.3622 |  |
| Gly1204 | c.3610 |  |  | **NC interruption (XXII)** | | |  | Gly1211 | c.3631 |  |
| Gly1207 | c.3619 |  |  | Gly1224 | c.3670 | B |  | Gly1214 | c.3640 |  |
| Gly1210 | c.3628 |  |  | Gly1227 | c.3679 |  |  | Gly1217 | c.3649 |  |
| Gly1213 | c.3637 |  |  | Gly1230 | c.3688 |  |  | Gly1220 | c.3658 |  |
| Gly1216 | c.3646 |  |  | Gly1233 | c.3697 |  |  | Gly1223 | c.3667 |  |
| Gly1219 | c.3655 |  |  | Gly1236 | c.3706 | S |  | Gly1226 | c.3676 |  |
| Gly1222 | c.3664 |  |  | Gly1239 | c.3715 |  |  | Gly1229 | c.3685 |  |
| Gly1225 | c.3673 |  |  | Gly1242 | c.3724 |  |  | Gly1232 | c.3694 |  |
| Gly1228 | c.3682 |  |  | Gly1245 | c.3733 |  |  | Gly1235 | c.3703 |  |
| Gly1231 | c.3691 | B |  | Gly1248 | c.3742 | B |  | Gly1238 | c.3712 |  |
|  |  |  |  |  |  |  |  | Gly1241 | c.3721 |  |
|  |  |  |  |  |  |  |  | Gly1244 | c.3730 | B |
| **NC interruption (XXI)** | | |  | **NC interruption (XXIII)** | | |  | **NC interruption (XXI)** | | |
| Gly1236 | c.3706 | B |  | Gly1258 | c.3772 | B |  | Gly1249 | c.3745 | B |
| Gly1239 | c.3715 |  |  | Gly1261 | c.3781 |  |  | Gly1252 | c.3754 |  |
| Gly1242 | c.3724 |  |  | Gly1264 | c.3790 |  |  | Gly1255 | c.3763 |  |
| Gly1245 | c.3733 |  |  | Gly1267 | c.3799 |  |  | Gly1258 | c.3772 |  |
| Gly1248 | c.3742 |  |  | Gly1270 | c.3808 |  |  | Gly1261 | c.3781 |  |
| Gly1251 | c.3751 | S |  | Gly1273 | c.3817 | S |  | Gly1264 | c.3790 | S |
| Gly1254 | c.3760 |  |  | Gly1276 | c.3826 |  |  | Gly1267 | c.3799 | S |
| Gly1257 | c.3769 |  |  | Gly1279 | c.3835 |  |  | Gly1270 | c.3808 | S |
| Gly1260 | c.3778 | B |  | Gly1282 | c.3844 | B |  | Gly1273 | c.3817 |  |
| **NC interruption (XXII)** | | |  | **NC interruption (XXIV)** | | |  | Gly1276 | c.3826 |  |
| Gly1265 | c.3793 | B |  | Gly1289 | c.3865 | B |  | Gly1279 | c.3835 |  |
| Gly1268 | c.3802 |  |  | Gly1292 | c.3874 |  |  | Gly1282 | c.3844 |  |
| Gly1271 | c.3811 |  |  | Gly1295 | c.3883 |  |  | Gly1284 | c.3850 | Y |
| Gly1274 | c.3820 |  |  | Gly1298 | c.3892 |  |  | Gly1285 | c.3853 |  |
| Gly1277 | c.3829 |  |  | Gly1301 | c.3901 |  |  | Gly1288 | c.3862 |  |
| Gly1280 | c.3838 |  |  | Gly1304 | c.3910 |  |  | Gly1291 | c.3871 |  |
| Gly1283 | c.3847 |  |  | Gly1307 | c.3919 |  |  | Gly1294 | c.3880 |  |
| Gly1286 | c.3856 |  |  | Gly1310 | c.3928 |  |  | Gly1297 | c.3889 |  |
| Gly1289 | c.3865 |  |  | Gly1313 | c.3937 |  |  | Gly1300 | c.3898 |  |
| Gly1292 | c.3874 |  |  | Gly1316 | c.3946 |  |  | Gly1303 | c.3907 |  |
| Gly1295 | c.3883 | S |  | Gly1319 | c.3955 |  |  | Gly1306 | c.3916 |  |
| Gly1298 | c.3892 |  |  | Gly1322 | c.3964 |  |  | Gly1309 | c.3925 |  |
| Gly1301 | c.3901 |  |  | Gly1325 | c.3973 | S |  | Gly1312 | c.3934 |  |
| Gly1304 | c.3910 |  |  | Gly1328 | c.3982 |  |  | Gly1315 | c.3943 | S |
| Gly1307 | c.3919 |  |  | Gly1331 | c.3991 |  |  | Gly1318 | c.3952 |  |
| Gly1310 | c.3928 |  |  | Gly1334 | c.4000 |  |  | Gly1321 | c.3961 |  |
| Gly1313 | c.3937 |  |  | Gly1337 | c.4009 |  |  | Gly1324 | c.3970 |  |
| Gly1316 | c.3946 |  |  | Gly1340 | c.4018 |  |  | Gly1327 | c.3979 |  |
| Gly1319 | c.3955 | S |  | Gly1343 | c.4027 |  |  | Gly1330 | c.3988 |  |
| Gly1322 | c.3964 |  |  | Gly1346 | c.4036 |  |  | Gly1333 | c.3997 |  |
| Gly1325 | c.3973 |  |  | Gly1349 | c.4045 |  |  | Gly1336 | c.4006 |  |
| Gly1328 | c.3982 |  |  | Gly1352 | c.4054 |  |  | Gly1339 | c.4015 | S |
| Gly1331 | c.3991 |  |  | Gly1355 | c.4063 |  |  | Gly1342 | c.4024 |  |
| Gly1334 | c.4000 |  |  | Gly1358 | c.4072 |  |  | Gly1345 | c.4033 |  |
| Gly1337 | c.4009 |  |  | Gly1361 | c.4081 | S |  | Gly1348 | c.4042 |  |
| Gly1340 | c.4018 |  |  | Gly1364 | c.4090 | S |  | Gly1351 | c.4051 |  |
| Gly1343 | c.4027 | S |  | Gly1367 | c.4099 | B |  | Gly1354 | c.4060 |  |
| Gly1346 | c.4036 |  |  |  |  |  |  | Gly1357 | c.4069 |  |
| Gly1349 | c.4045 | B |  |  |  |  |  | Gly1360 | c.4078 |  |
|  |  |  |  |  |  |  |  | Gly1363 | c.4087 | S |
|  |  |  |  |  |  |  |  | Gly1366 | c.4096 |  |
|  |  |  |  |  |  |  |  | Gly1369 | c.4105 |  |
|  |  |  |  |  |  |  |  | Gly1372 | c.4114 | B |
| **NC interruption (XXIII)** | | |  | **NC interruption (XXV)** | | |  | **NC interruption (XXII)** | | |
| Gly1358 | c.4072 | B |  | Gly1380 | c.4138 | B |  | Gly1379 | c.4135 | B |
| Gly1361 | c.4081 |  |  | Gly1383 | c.4147 |  |  | Gly1382 | c.4144 |  |
| Gly1364 | c.4090 |  |  | Gly1386 | c.4156 |  |  | Gly1385 | c.4153 |  |
| Gly1367 | c.4099 |  |  | Gly1389 | c.4165 |  |  | Gly1388 | c.4162 |  |
| Gly1370 | c.4108 |  |  | Gly1392 | c.4174 |  |  | Gly1391 | c.4171 |  |
| Gly1373 | c.4117 |  |  | Gly1395 | c.4183 |  |  | Gly1394 | c.4180 |  |
| Gly1376 | c.4126 |  |  | Gly1398 | c.4192 |  |  | Gly1397 | c.4189 |  |
| Gly1379 | c.4135 |  |  | Gly1401 | c.4201 |  |  | Gly1400 | c.4198 |  |
| Gly1382 | c.4144 |  |  | Gly1404 | c.4210 | B |  | Gly1403 | c.4207 |  |
| Gly1385 | c.4153 | S |  | **NC interruption (XXVI)** | | |  | Gly1406 | c.4216 | S |
| Gly1388 | c.4162 |  |  | Gly1406 | c.4216 | B, S |  | Gly1409 | c.4225 |  |
| Gly1391 | c.4171 |  |  | Gly1409 | c.4225 |  |  | Gly1412 | c.4234 |  |
| Gly1394 | c.4180 |  |  | Gly1412 | c.4234 |  |  | Gly1415 | c.4243 |  |
| Gly1397 | c.4189 |  |  | Gly1415 | c.4243 |  |  | Gly1418 | c.4252 |  |
| Gly1400 | c.4198 |  |  | Gly1418 | c.4252 |  |  | Gly1421 | c.4261 |  |
| Gly1403 | c.4207 |  |  | Gly1421 | c.4261 |  |  | Gly1424 | c.4270 |  |
| Gly1406 | c.4216 |  |  | Gly1424 | c.4270 |  |  | Gly1426 | c.4276 | Y |
| Gly1409 | c.4225 |  |  | Gly1427 | c.4279 |  |  | Gly1427 | c.4279 |  |
| Gly1412 | c.4234 |  |  | Gly1430 | c.4288 |  |  | Gly1430 | c.4288 |  |
| Gly1415 | c.4243 |  |  | Gly1433 | c.4297 |  |  | Gly1433 | c.4297 |  |
| Gly1418 | c.4252 | S |  | Gly1436 | c.4306 |  |  | Gly1436 | c.4306 |  |
| Gly1421 | c.4261 |  |  | Gly1439 | c.4315 |  |  | Gly1439 | c.4315 | S |
| Gly1424 | c.4270 |  |  | Gly1442 | c.4324 |  |  | Gly1442 | c.4324 |  |
| Gly1427 | c.4279 |  |  | Gly1443 | c.4327 | X |  | Gly1445 | c.4333 |  |
| Gly1430 | c.4288 |  |  | Gly1445 | c.4333 | S |  | Gly1448 | c.4342 |  |
| Gly1433 | c.4297 |  |  | Gly1448 | c.4342 |  |  | Gly1451 | c.4351 |  |
| Gly1436 | c.4306 | B |  | Gly1451 | c.4351 |  |  | Gly1454 | c.4360 |  |
|  |  |  |  | Gly1454 | c.4360 |  |  | Gly1457 | c.4369 |  |
|  |  |  |  | Gly1457 | c.4369 | B |  | Gly1460 | c.4378 | B |
| **Carboxy NC domain** | | |  | **Carboxy NC domain** | | |  | **Carboxy NC domain** | | |
| Gly1445 | c.4333 | NC |  | Gly1459 | c.4375 | NC |  | Gly1467 | c.4399 | NC |
| Gly1463 | c.4387 | NC |  | Gly1461 | c.4381 | NC |  | Gly1485 | c.4453 | NC |
| Gly1470 | c.4408 | NC |  | Gly1464 | c.4390 | NC |  | Gly1492 | c.4474 | NC |
| Gly1478 | c.4432 | NC |  | Gly1465 | c.4393 | NC |  | Gly1500 | c.4498 | NC |
| Gly1484 | c.4450 | NC |  | Gly1483 | c.4447 | NC |  | Gly1506 | c.4516 | NC |
| Gly1488 | c.4462 | NC, S |  | Gly1490 | c.4468 | NC |  | Gly1510 | c.4528 | NC, S |
| Gly1491 | c.4471 | NC |  | Gly1498 | c.4492 | NC |  | Gly1513 | c.4537 | NC |
| Gly1538 | c.4612 | NC |  | Gly1508 | c.4522 | NC, S |  | Gly1560 | c.4678 | NC |
| Gly1553 | c.4657 | NC |  | Gly1511 | c.4531 | NC |  | Gly1595 | c.4783 | NC |
| Gly1573 | c.4717 | NC |  | Gly1598 | c.4792 | NC |  | Gly1602 | c.4804 | NC |
| Gly1580 | c.4738 | NC |  | Gly1606 | c.4816 | NC |  | Gly1612 | c.4834 | NC |
| Gly1590 | c.4768 | NC |  | Gly1608 | c.4822 | NC |  | Gly1615 | c.4843 | NC |
| Gly1593 | c.4777 | NC |  | Gly1611 | c.4831 | NC |  | Gly1617 | c.4849 | NC |
| Gly1595 | c.4783 | NC |  | Gly1612 | c.4834 | NC |  | Gly1624 | c.4870 | NC |
| Gly1602 | c.4804 | NC |  | Gly1613 | c.4837 | NC |  | Gly1640 | c.4918 | NC |
| Gly1618 | c.4852 | NC |  | Gly1620 | c.4858 | NC |  | Gly1642 | c.4924 | NC |
| Gly1620 | c.4858 | NC |  | Gly1636 | c.4906 | NC |  | Gly1675 | c.5023 | NC |
| Gly1653 | c.4957 | NC |  | Gly1639 | c.4915 | NC |  |  |  |  |

**Supplemental Table 3.** Logistic regression model of molecular characteristics of *COL4A3* and *COL4A4* Gly missense variants associated with haematuria in the 100kGP database, excluding two common *COL4A3* variants Gly695Arg and Gly1277Ser.

|  | Estimate (SE) | p-value |
| --- | --- | --- |
| **a) All variants (n=253 individuals)** | | |
| Intercept | -2.31 (0.49) | **<0.001** |
| Location in exons 21+ | 0.36 (0.38) | 0.336 |
| Location adjacent to NC region | -1.35 (0.63) | **0.031** |
| Substitution with Arg/Val/Glu/Asp/Trp | 0.82 (0.44) | 0.064 |
| *Pseudo-R^2^_McFadden_ = 0.043* | | |
| **b) Excluding NC boundary variants (n=207 individuals)** | | |
| Intercept | -2.37 (0.52) | **<0.001** |
| Location in exons 21+ | 0.24 (0.39) | 0.543 |
| Location at amino end of local collagenous region | 0.47 (0.42) | 0.265 |
| Location at carboxyl end of local collagenous region | 0.99 (0.49) | **0.041** |
| Substitution with Arg/Val/Glu/Asp/Trp | 0.66 (0.46) | 0.149 |
| *Pseudo-R^2^_McFadden_ = 0.039* | | |

NC, Non-collagenous; SE, Standard error.


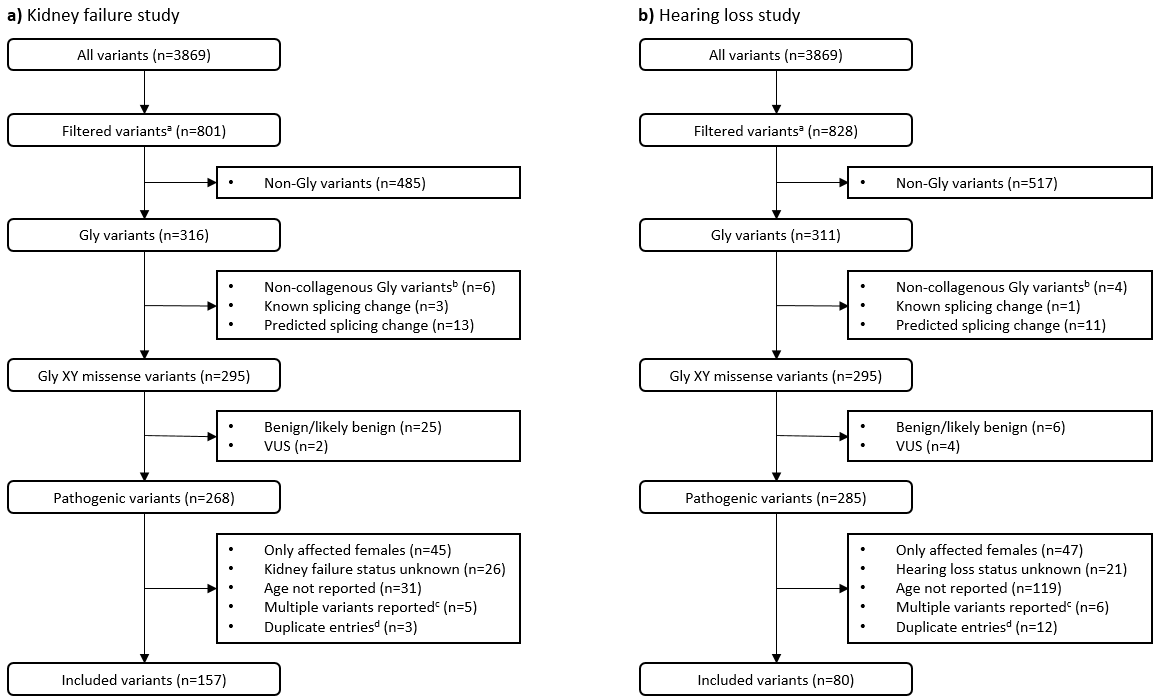


**Supplemental Figure 1.** Variant inclusion flowcharts for the **(a)** kidney failure and **(b)** hearing loss study cohorts.

VUS, Variant of uncertain significance.

^a^ Initial filtering process described in text.

^b^ Gly missense variants located within non-collagenous interruptions or termini.

^c^ Individuals found with more than one variant across any of the *COL4A3* – *COL4A5* genes.

^d^ Including where individuals from the same family were listed as separate entries.


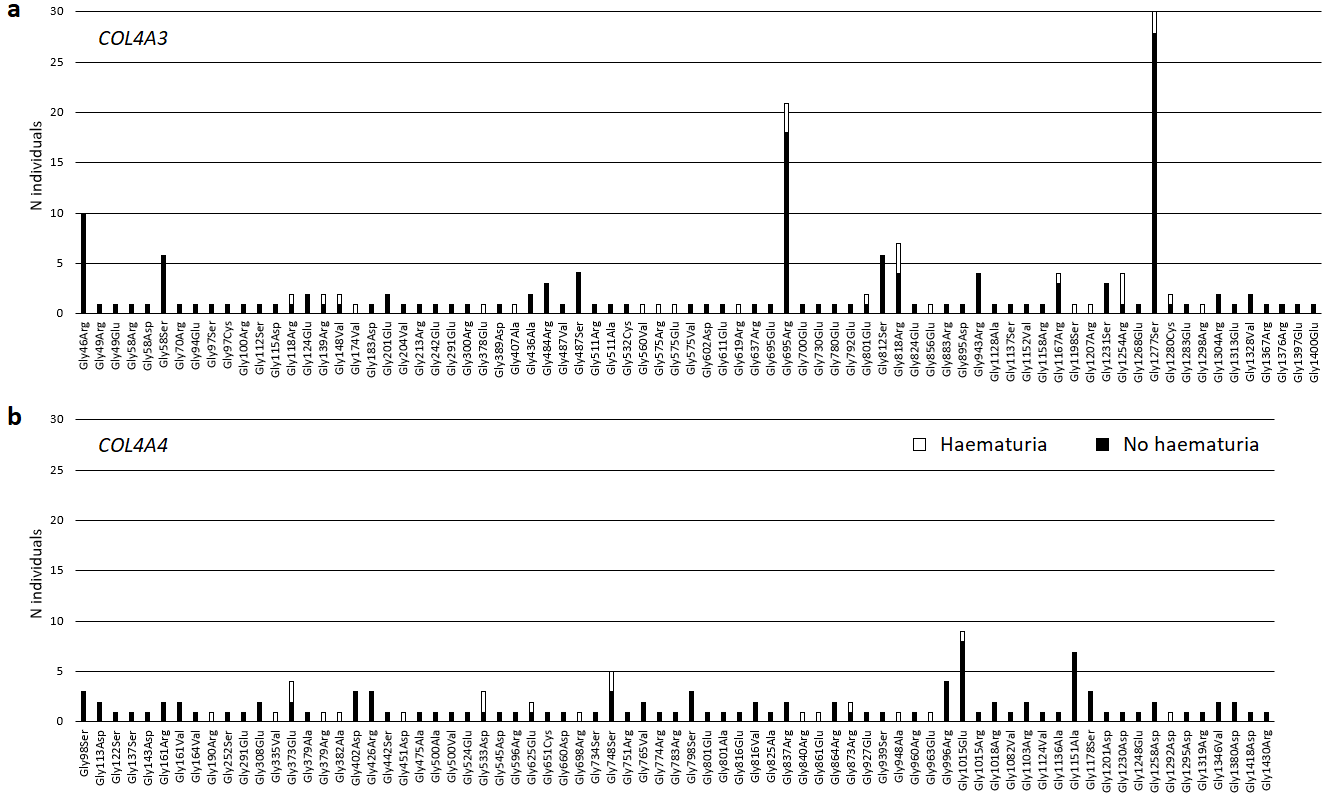


**Supplemental Figure 2.** **(a)** *COL4A3* and **(b)** *COL4A4* Gly missense variants reported in individuals with and without haematuria in the 100kGP database.
